# Plant begomoviruses subvert ubiquitination to suppress plant defenses against insect vectors

**DOI:** 10.1371/journal.ppat.1007607

**Published:** 2019-02-21

**Authors:** Ping Li, Chao Liu, Wen-Hao Deng, Dan-Mei Yao, Li-Long Pan, Yun-Qin Li, Yin-Quan Liu, Yan Liang, Xue-Ping Zhou, Xiao-Wei Wang

**Affiliations:** 1 Ministry of Agriculture Key Lab of Molecular Biology of Crop Pathogens and Insects, Institute of Insect Sciences, Zhejiang University, Hangzhou, China; 2 Center of Analysis and Measurement, Zhejiang University, Hangzhou, China; 3 Institute of Biotechnology, Zhejiang University, Hangzhou, China; University of California Davis, UNITED STATES

## Abstract

Most plant viruses are vectored by insects and the interactions of virus-plant-vector have important ecological and evolutionary implications. Insect vectors often perform better on virus-infected plants. This indirect mutualism between plant viruses and insect vectors promotes the spread of virus and has significant agronomical effects. However, few studies have investigated how plant viruses manipulate plant defenses and promote vector performance. Begomoviruses are a prominent group of plant viruses in tropical and sub-tropical agro-ecosystems and are transmitted by whiteflies. Working with the whitefly *Bemisia tabaci*, begomoviruses and tobacco, we revealed that C2 protein of begomoviruses lacking DNA satellites was responsible for the suppression of plant defenses against whitefly vectors. We found that infection of plants by tomato yellow leaf curl virus (TYLCV), one of the most devastating begomoviruses worldwide, promoted the survival and reproduction of whitefly vectors. TYLCV C2 protein suppressed plant defenses by interacting with plant ubiquitin. This interaction compromised the degradation of JAZ1 protein, thus inhibiting jasmonic acid defense and the expression of MYC2-regulated terpene synthase genes. We further demonstrated that function of C2 protein among begomoviruses not associated with satellites is well conserved and ubiquitination is an evolutionarily conserved target of begomoviruses for the suppression of plant resistance to whitefly vectors. Taken together, these results demonstrate that ubiquitination inhibition by begomovirus C2 protein might be a general mechanism in begomovirus, whitefly and plant interactions.

## Author summary

In the long evolutionary history, plants, vector-borne viruses and insects have built complicated relationships. A growing body of evidence has shown that virus infection of the plant can influence vector physiology and behavior to increase virus transmission. However, the underlying mechanisms are largely unknown. Working with begomoviruses, a group of over 300 single-stranded plant DNA virus species, which are transmitted by the whitefly *Bemisia tabaci* and have caused extensive damage to many crops worldwide, we addressed whether and how begomovirus infection suppresses plant defense responses to insect vectors. We found that begomovirus C2 protein was responsible for the suppression of plant defenses and contributed to the virus–vector mutualism. Viral C2 directly interacted with plant ubiquitin to compromise the degradation of JAZ1 protein and therefore inhibited the expression of down-stream defense genes. We further demonstrated that function of C2 was well conserved among begomoviruses for the suppression of plant resistance to vectors. This study also shows how sophisticated mutualism has evolved in the begomovirus-insect vector system. Our findings expand fascinating new horizons in the field of research on the interactions among plants, vectors and viruses and might lead to new strategies to combat begomovirus spread.

## Introduction

Vector-borne viruses and their insect vectors have coevolved complex relationships [1–4]. On the one hand, viruses and their vectors form a competitive relationship because they share the same host plants. On the other hand, due to the immobility of the host plant, insect vectors play significant roles in the epidemiology of plant viruses [5–10]. Attracting vectors to infected plants, facilitating their feeding and population growth and then dispersal of vectors carrying viruses to new plants would be highly beneficial to virus spread [11–13]. Several animal pathogens can directly affect their vectors to increase transmission rate [14]. By contrast, plant pathogens have been shown mainly to modify behavior of vectors via their shared host plant to achieve an indirect mutualistic relationship between pathogen and vector [15]. However, the innermost mechanisms of such mutualisms are largely unknown.

Geminiviruses are a prominent group of plant viruses in tropical and sub-tropical agro-ecosystems worldwide that cause significant damage to agricultural production [16,17]. Geminiviruses are classified into 9 genera: *Mastrevirus*, *Topocuvirus*, *Curtovirus*, *Becurtovirus*, *Eragrovirus*, *Turncurtovirus*, *Grablovirus*, *Capulavirus*, and *Begomovirus* [18]. The genus *Begomovirus* is the largest in the family, with more than 300 accepted species. Geminivirus genomes can be bipartite or monopartite [19]. Genomes of bipartite geminiviruses contain two circular ssDNA components: DNA-A and DNA-B. By contrast, genomes of monopartite geminiviruses possess only one component resembling DNA-A. The genome size of begomovirus DNA-A is around 2,800 nt, which harbors two genes (V1, coat protein and V2) on the virion-sense strand and four genes (replication initiation protein [Rep, also known as C1]; transcription activator protein [TrAP, C2]; replication enhancer protein [C3] and C4) on the complementary strand [20,21]. In some instances, geminiviruses are accompanied by circular ssDNA alphasetallites, betasatellites or deltasatellites [22]. Interestingly, among the 9 genera, only some members of the genus *Begomovirus* contain DNA-B or are associated with betasatellites that encode the pathogenic factor βC1 [19]. The genome of viruses in the other 8 genera and many begomoviruses only consist of one circular single stranded DNA molecule similar to DNA-A [19]. Geminiviruses are transmitted by insect vectors and members of the genus *Begomovirus* are exclusively transmitted by whiteflies of the *Bemisia tabaci* complex in a persistent manner [7,23–26]. Plant-mediated interactions between geminiviruses and insect vectors exert important influences on both the distribution and abundance of the vector insects and epidemiology of geminivirus diseases [13,27]. During the last decade, a few studies have explored the interactions between plants, geminiviruses and whiteflies [28–30]. However, these studies only investigated tomato yellow leaf curl China virus (TYLCCNV), a begomovirus associated with a betasatellite (TYLCCNV betasatellite, TYLCCNB) [31]. These results have shown that βC1 protein encoded by TYLCCNB plays a key role in suppression of plant jasmonic acid (JA) resistance to promote the performance of its vector insects [32,33]. However, little is known about whether and how infection of monopartite begomoviruses in the absence of a betasatellite affects the performance of whiteflies.

JA plays important roles in plant defense against insects. Whitefly nymphs develop slower and have a lower survival rate after JA treatments [34]. Zhang *et al*. reported that transgenic tomato with impaired JA defenses (*spr-2* and *def-1*) promotes whitefly nymphal development [35]. Furthermore, another study demonstrates that *B*. *tabaci* deposits more eggs on *spr2* tomato plants [36]. Interestingly, begomovirus C2 has been reported to affect plant JA signaling pathway as well. In leaves of transgenic plants expressing African cassava mosaic virus C2, genes related to JA biosynthesis were up-regulated [37]. C2 of *Tomato yellow leaf curl Sardinia virus* (TYLCSV) specifically affects JA-induced responses by interacting with CSN5 and plants expressing C2 are more susceptible to pathogen attack [38]. Recent results have showed that the monopartite begomovirus, tomato yellow leaf curl virus (TYLCV), infection also affects reproduction and survival of whiteflies and this is associated with inhibition of JA pathway [39]. Based on these findings, a key question was raised: how monopartite begomoviruses not associated with betasatellite or DNA B suppress plant defense and affect whitefly survival? Since bipartite begomoviruses are supposed to have evolved from monopartite viruses by capturing an ancestor of what is today called a DNA-B [22], answering this question will help us to explain how sophisticated mutualism has arisen in the begomovirus-insect vector system.

In this study, we elucidated the mechanisms of plant-mediated mutualism between whiteflies and begomoviruses in the absence of any DNA satellite via an integration of ecological and molecular approaches. We found that infection of TYLCV, one of the most devastating begomoviruses usually not associated with a betasatellite [40,41], promoted the performance of whiteflies in tobacco. Further studies revealed that C2 protein of TYLCV suppressed plant defense via interaction with ubiquitin-40S ribosomal protein S27a (RPS27A). Next, we showed that C2 from another monopartite begomovirus, papaya leaf curl China virus (PaLCuCNV), could promote the performance of their vectors as well and interaction between C2 and RPS27A protein was conserved among tobacco, tomato and *Arabidopsis*. Interestingly, C2 of TYLCCNV could not interact with RPS27A, which may partially elucidate the reason why TYLCCNV with mutant TYLCCNB cannot promote the performance of whitefly. Overall, our findings reveal an evolutionarily conserved strategy for hijacking plant defense by begomoviruses not associated with betasatellites.

## Results

### TYLCV infection of host plants benefits whitefly vectors by suppressing JA signaling pathway

**Fig 1:**
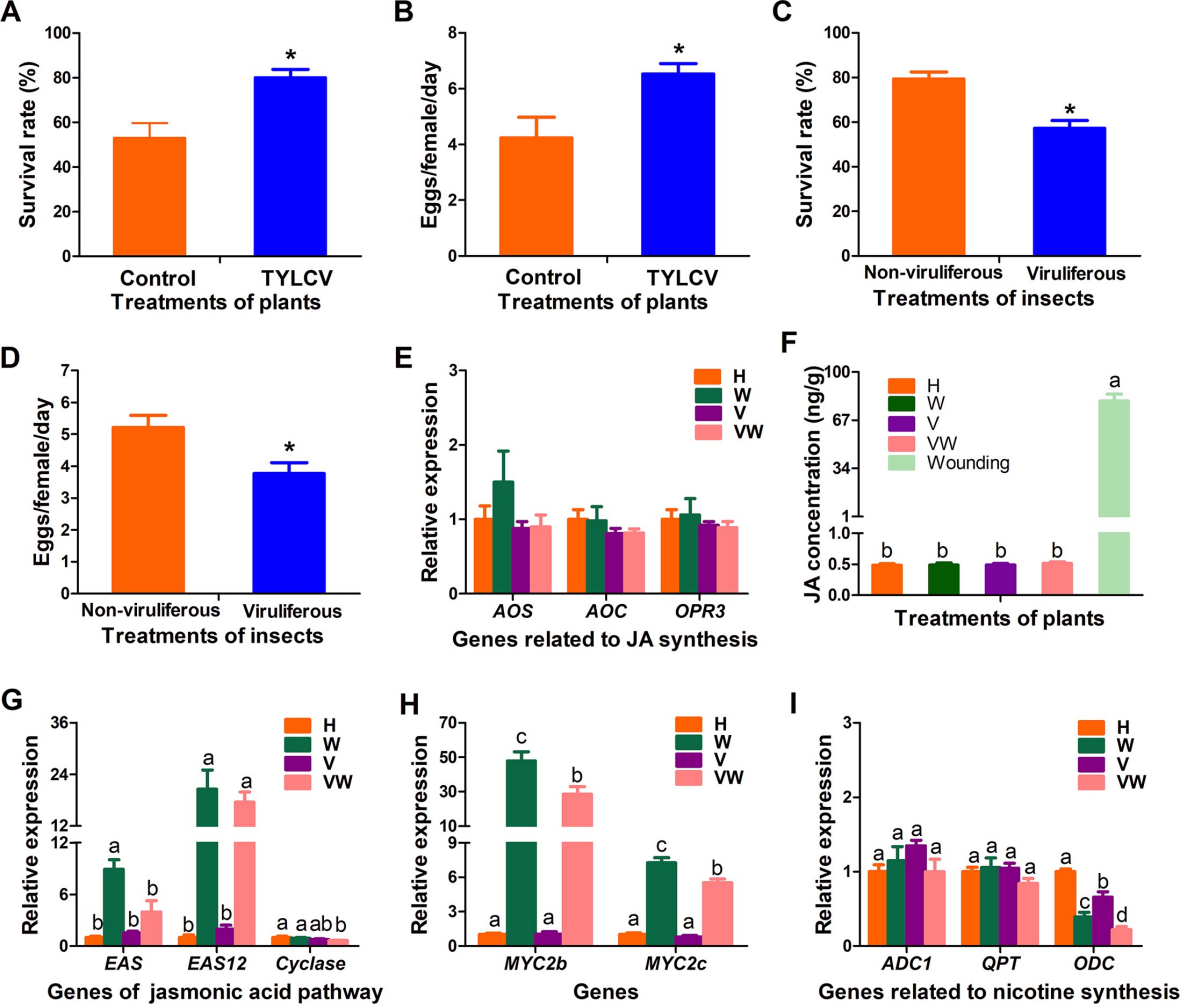
TYLCV infection subverts plant JA signaling pathway to benefit whitefly. (A) Survival rate of adult whitefly on control and TYLCV-infected tobacco plants. Values are means±SE, n = 30. (B) Daily number of eggs laid per female whitefly on control and TYLCV-infected tobacco plants. Values are means±SE, n = 30. (C) Survival rate of non-viruliferous and viruliferous adult whiteflies on cotton plants. Values are means±SE, n = 30. (D) Daily number of eggs laid per non-viruliferous and viruliferous adult female whiteflies on cotton plants. Values are means±SE, n = 30. (E) Expression of JA synthesis-related genes [*allene oxide synthase* (*AOS*), *allene oxide cyclase* (*AOC*) and *12-oxophytodienoate reductase 3* (*OPR3*)] in healthy (H), whitefly infestation (W), TYLCV infection (V) plants and plants with both TYLCV and whiteflies (VW). Values are means±SE, n = 8. (F) JA content in tobacco plants after different treatments: healthy (H), whitefly infestation (W), TYLCV infection (V) plants and plants with both TYLCV and whiteflies (VW) and wounding plants. Values are means±SE, n>6. (G) Expression of genes related to terpene syntheses [*epi-arisotolchene synthase* (*EAS*), *epi-arisotolchene syntheses 12* (*EAS12*), *terpenoid cyclase* (*cyclase*)] in healthy (H), whitefly infestation (W), TYLCV infection (V) plants and plants with both TYLCV and whiteflies (VW). Values are means±SE, n = 8. (H) Expression levels of *MYC2* in healthy (H), whitefly infestation (W), TYLCV infection (V) plants and plants with both TYLCV and whiteflies (VW). Values are means±SE, n = 8. (I) Expression level of genes related to nicotine syntheses in healthy (H), whitefly infestation (W), TYLCV infection (V) plants and plants with both TYLCV and whiteflies (VW). Values are means±SE, n = 8. Asterisks or different letters above the bars indicate significant differences between different treatments (P < 0.05; Student’s t test for all experiments). All experiments were repeated three times with similar results.

The TYLCV-tobacco-whitefly system was used to investigate whether monopartite begomovirus infection on plants affects vector performance. Seven days after the release of whiteflies onto the plants, the survival and fecundity of whiteflies on TYLCV-infected plants were significantly higher than those on uninfected plants (Fig 1A & 1B). The enhanced whitefly performance on TYLCV-infected plants could be due to direct effects of viral infection on insects, or viral suppression of plant defense that indirectly promote insect performance. Whitefly performance was significantly reduced when the viruliferous whiteflies were transferred to cotton plants, a non-host of TYLCV (Fig 1C & 1D), indicating that virus acquisition did not promote but reduced whitefly performance. Taken together, these results indicate that TYLCV infection suppresses plant defense and in turn benefits its insect vector whitefly.

To investigate how TYLCV infection regulates plant defenses, we monitored the expression of genes related to jasmonic acid (JA) synthesis. Expressions of three JA biosynthesis genes; *allene oxide synthase* (*AOS*), *allene oxide cyclase* (*AOC*) and *12-oxophytodienoate reductase 3* (*OPR3*) and JA content were not affected by virus infection (Fig 1E & 1F). Synthesis of some JA-regulated secondary metabolites, such as terpenes, is one of the major mechanisms by which plants defend themselves against the insects and TYLCCNB has been reported to suppress plant defense against whitefly by reducing terpene production [27,33]. Thus, we examined the expression of genes related to terpene synthesis, including epi-arisotolchene synthase (*EAS*), EAS12 and terpenoid cyclase (*Cyclase*). The transcript levels of *EAS* and *Cyclase* genes were significantly decreased in the virus and whitefly co-infection plants compared to whitefly infested plants (Fig 1G). As *EAS* has been verified to be associated with tobacco defense against whiteflies previously [27], we examined whether another gene *Terpene Cyclase* is responsible for plant defense against whiteflies by virus-induced gene silencing. Our results demonstrated that silencing of *Cyclase* significantly increased whitefly fecundity but not the survival rate (S1A–S1C Fig).

MYC2 is reported to be a major transcript factor responsible for JA-regulated secondary metabolites synthesis [33,42,43]. Sequence alignment from NCBI showed there are 3 MYC2 genes (*MYC2a*, *MYC2b* and *MYC2c*) in tobacco and their sequences are highly homologous. Therefore, we determined the expression of *MYC2a/b* and *MYC2c* after different treatments. As shown in Fig 1H, compared to whitefly infestation, expression level of MYC2 in plants which had both TYLCV and whiteflies was reduced, which implies that down-stream JA signaling pathway was hijacked by TYLCV. Bioassay of whitefly on *myc2*-silencing plants also indicated that MYC2 is a key factor to regulate plant defense (S1D–S1F Fig). As to nicotine, another JA-regulated secondary metabolite, although the expression of ornithine decarboxylase (*ODC*) was decreased, compared to controls there were no significant differences in expression levels of *arginine decarboxylase1* (*ADC1*) and quinolinic acid phosphoribosyltransferase (*QPT*) genes (Fig 1I). Further study showed that *ODC* might not be a factor responsible for plant defense against whiteflies (S1G–S1I Fig). Taken together, TYLCV infection does not affect plant JA biosynthesis but appears to subvert parts of downstream JA signaling pathway and in turn promotes whitefly performance.

### TYLCV C2 is a factor for virus-induced promotion of whitefly performance

**Fig 2:**
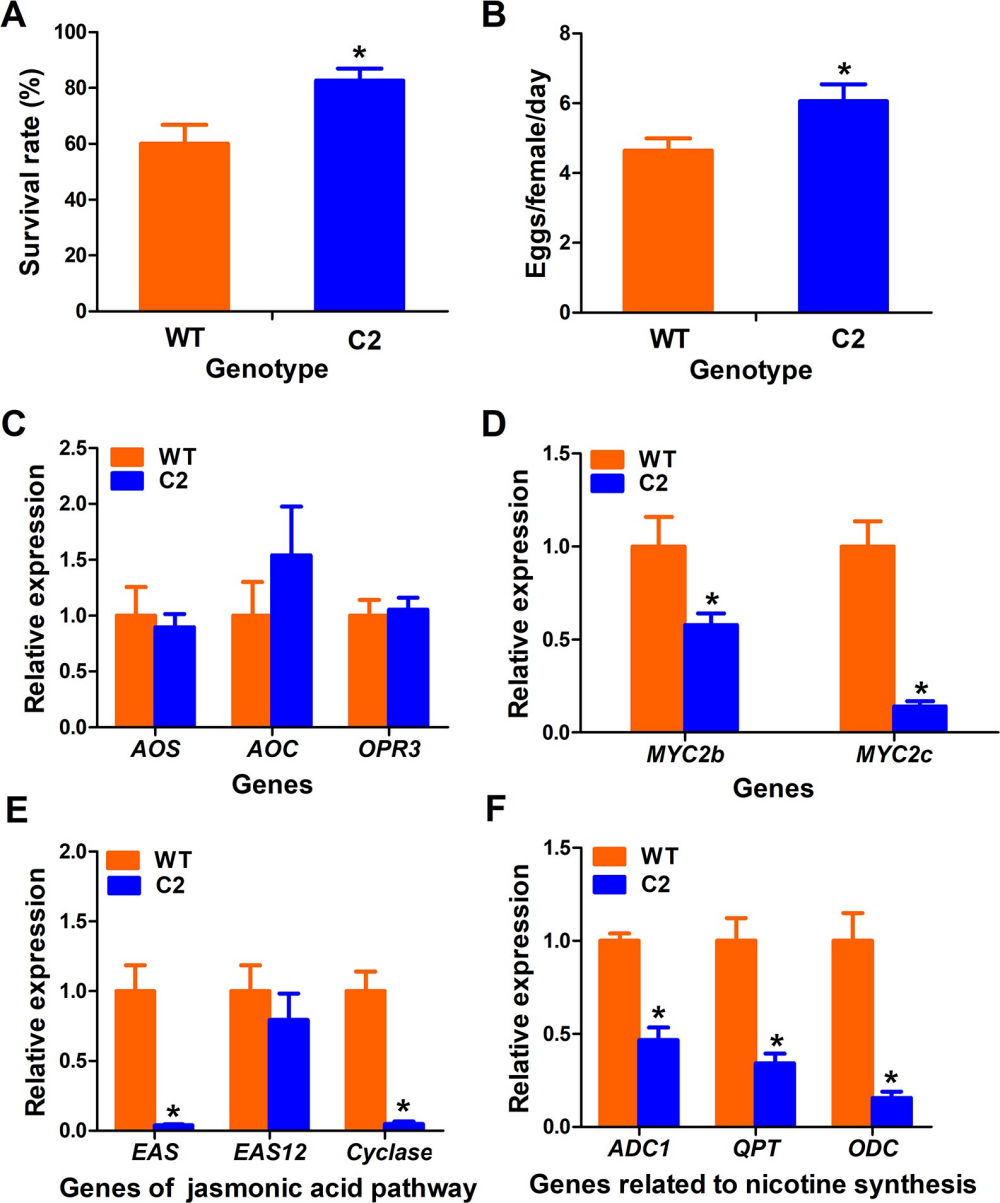
TYLCV C2 promotes whitefly performance by suppressing plant JA signaling. (A) Survival rate of adult whitefly on wild type tobacco plants (WT) and the transgenic C2 expressing tobacco plants (C2). Values are means±SE, n = 30. (B) Daily number of eggs laid by per female whitefly on wild type tobacco plants and C2 expressing tobacco plants. Values are means±SE, n = 30. (C) Expression of genes related to JA synthesis [*allene oxide synthase* (*AOS*), *allene oxide cyclase* (*AOC*) and *12-oxophytodienoate reductase 3* (*OPR3*)] in wild type (WT) and C2 expressing tobacco plants. Values are means±SE, n = 8. (D) Expression level of *MYC2* in wild type (WT) and C2 expressing tobacco plants. Values are means±SE, n = 8. (E) Expression of genes related to terpene synthesis [*epi-arisotolchene synthase* (*EAS*), *epi-arisotolchene synthase 12* (*EAS12*), *terpenoid cyclase* (*cyclase*)] in wild type (WT) and C2 expressing tobacco plants. Values are means±SE, n = 8. (F) Expression levels of genes related to nicotine syntheses in wild type (WT) and C2 expressing tobacco plants. Values are means±SE, n = 8. Asterisks indicate significant differences between different treatments (*P* < 0.05; Student’s t test for all experiments). All experiments were repeated three times with similar results.

TYLCV genome only encodes 6 proteins, of which C1, C3 proteins are responsible for viral replication and V1 encodes the coat protein [44]. Previous studies have shown that C2, V2 and C4 proteins are the major factors in viral suppression of plant defenses [38,45,46]. To determine which of the three proteins regulates plant defense against whiteflies, we generated transgenic plants with ectopic expression of TYLCV C2, V2 or C4, respectively. Note that while C2 and V2 transgenic plants had no morphological differences with the wild type, the ectopic expression of C4 protein induced downward leaf curling in tobacco (S2A Fig). Bioassays showed that whiteflies survived better and laid more eggs on C2 transgenic plants than on wild-type (Fig 2A & 2B). However, performance of whiteflies on C4 or V2 transgenic plants was not significantly different from that on wild type (S2B–S2E Fig), suggesting that TYLCV C2 mediates the suppression of plant defense against whiteflies.

Next, we examined whether ectopic expression of C2 would affect JA signaling pathway. qRT-PCR analyses indicated that ectopic expression of C2 had no effects on expression levels of JA synthesis genes: *AOS*, *AOC* and *OPR3* but significantly suppressed the expression levels of *MYC2*, *EAS* and *Cyclase*, which are essential for terpene synthesis (Fig 2C–2E). In addition, ectopic expression of C2 suppressed the expression of genes related to nicotine synthesis (Fig 2F). Notably, different expression patterns of *ADC1*, *QPT* and *ODC* were observed between TYLCV infection and ectopic expression of C2 plants (Figs 1I & 2F), which may be due to different amount of C2 protein between TYLCV infected and transgenic plants. Taken together, our results demonstrate that TYLCV-induced promotion of whitefly performance depends on C2 protein through suppression of plant JA signaling pathway.

### C2 interacts with RPS27A *in vitro* and *in vivo*

**Fig 3:**
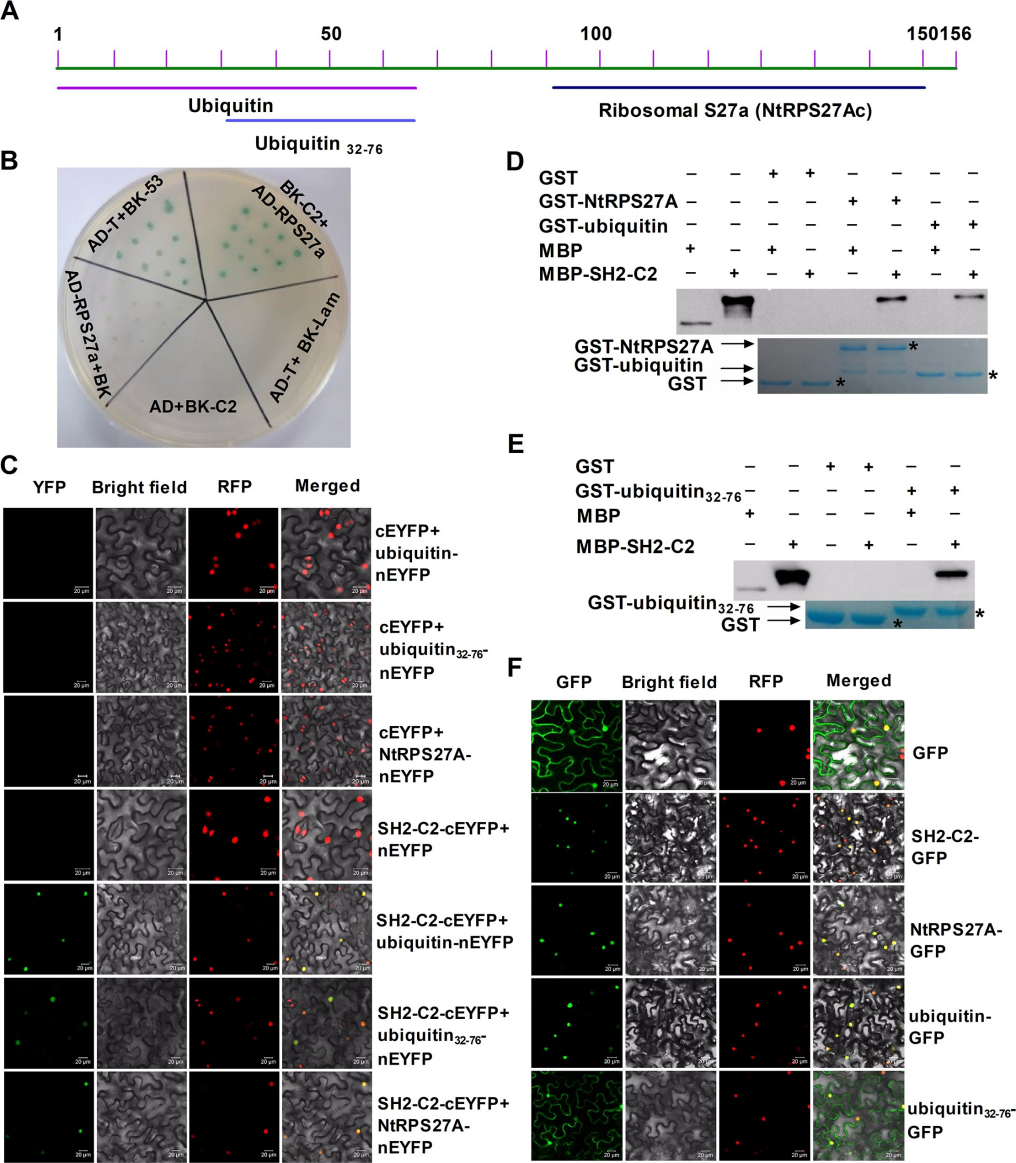
Localization of C2 and NtRPS27A and their interaction. (A) Structure of RPS27A. (B) Interaction between SH2-C2 and NtRPS27A in the yeast two-hybrid system. Yeast strain Y2H Gold co-transformed with the indicated plasmids was spotted on synthetic medium SD-Leu-Trp-His with x-α-gal and 2 mM 3-amino-1,2,4-triazole. The empty vectors pGBKT7 and pGADT7 were used as negative controls. (C) *In vivo* BiFC analysis of SH2-C2 interaction with NtRPS27A. Nuclei of tobacco leaf epidermal cells were marked with a RFP fusion protein that is located in Nuclei. Bars = 20 mm. (D) and (E) *In vitro* GST pull-down assays. MBP or MBP-SH2-C2 fusion proteins were pull-down by GST, GST-NtRPS27A, GST-ubiquitin or GST-ubiquitin_32-76_ fusion proteins. GST beads were washed and proteins were analyzed by SDS-PAGE western blot. Associated proteins were detected by anti-MBP antibody and gels were stained with Coomassie Brilliant Blue to monitor GST and GST fusion proteins. (F) Subcellular localization of SH2-C2 and different segments of NtRPS27A. Nuclei of tobacco leaf epidermal cells were marked with a RFP fusion protein that is located in Nuclei. Bars = 20 mm.

To elucidate how TYLCV C2 manipulates plant defense, we screened a tobacco cDNA library by yeast two-hybrid to identify plant proteins that interact with C2. We designed a yeast expressing vector containing 1–78 amino acid of C2 reported to be absent of transcriptional activation domain [47]. After yeast two-hybrid screening, a candidate protein *N*. *tabacum* RPS27A (NtRPS27A), which consists of a ubiquitin domain at the N terminus and a ribosomal protein S27a domain at the C terminus, was discovered (Fig 3A). With this candidate protein, we first confirmed the interaction between C2 and NtRPS27A in yeast two-hybrid (Fig 3B). Next, we performed a bimolecular fluorescence complementation (BiFC) assay to examine the *in vivo* interactions of C2 and NtRPS27A. Strong fluorescence was detected in leaves in which NtRPS27A and C2 were co-expressed (Fig 3C). Then we performed GST pull-down assays and confirmed the direct interaction between the two full-length proteins (Fig 3D). RPS27A contains two functional domains (Fig 3A). To determine which domain is responsible for the interaction, we performed BIFC and GST-pull down assays with truncated RPS27A. Our results showed that the ubiquitin domain rather than the C-terminal RPS27a domain (named RPS27Ac) could interact with C2, indicating a role of the ubiquitin in the interaction (Fig 3C and 3D & S3 Fig). As the RPS27A protein we screened by yeast two-hybrid is a truncated version only containing 32–76 amino acids of NtRPS27A (ubiquitin_32-76_), we speculated that this segment may determine the interaction between C2 and NtRPS27A. BiFC and GST pull-down assays ascertained that ubiquitin_32-76_ indeed interacted with C2 (Fig 3C & 3E). Moreover, we detected the intracellular localization of C2 and different segments of NtRPS27A proteins using GFP fusion by transient expression system. C2, NtRPS27A and ubiquitin were located in the nucleus (Fig 3F), whereas peptides containing 32–76 amino acid of ubiquitin were located in nucleus and cytosol, similar to free GFP (Fig 3F). Considered together, these results confirm that C2 interacts with NtRPS27A, and amino-acids 32–76 of ubiquitin domain in NtRPS27A are important for the interaction.

### *RPS27A* plays a role in tobacco defense against whiteflies

**Fig 4:**
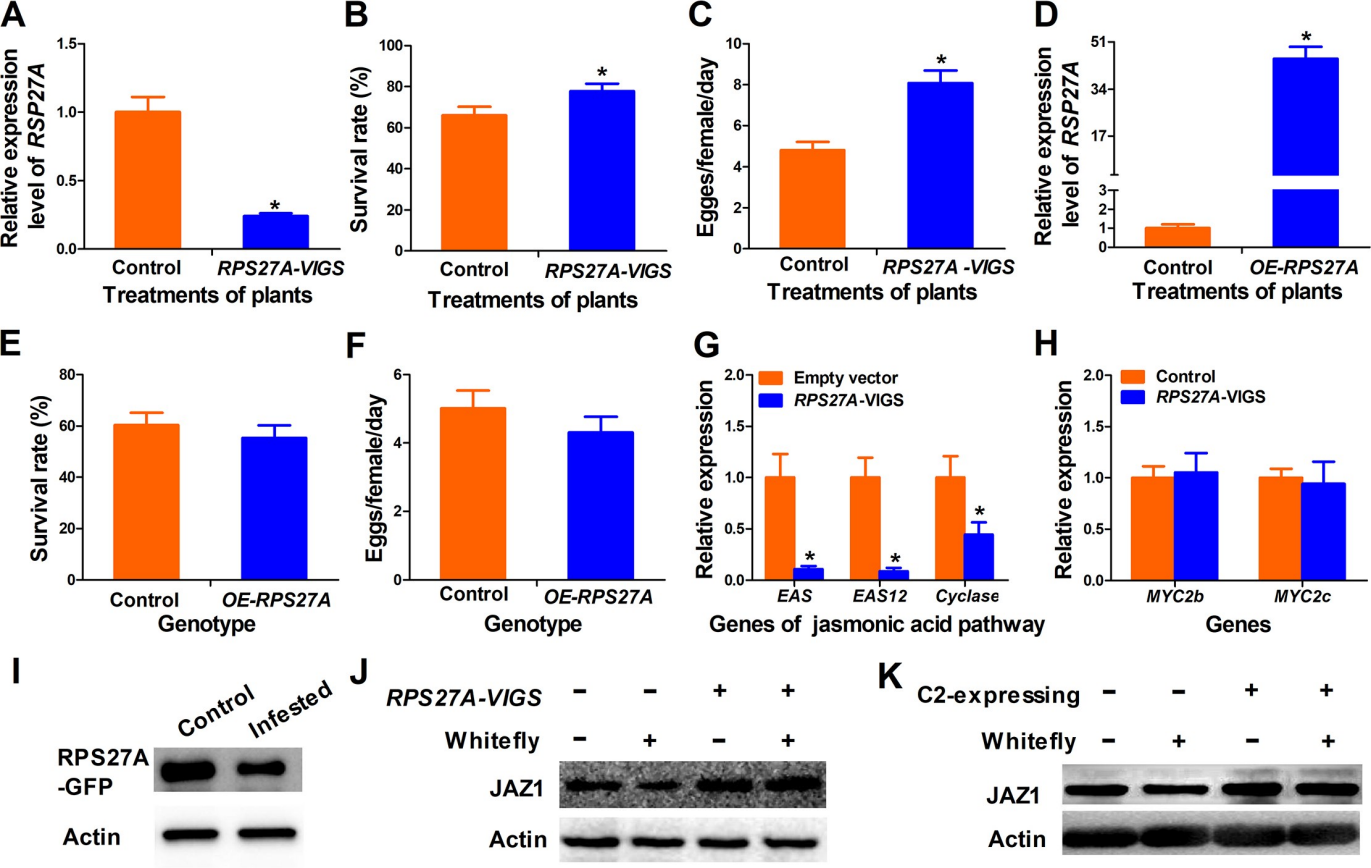
RPS27A and C2 protein regulate JAZ1 degradation to manipulate plant defense. (A) Expression of *RPS27A* gene in *RPS27A*-silencing tobacco plants. Values are means±SE, n = 8. (B) Survival rate of whitefly on control and *rps27A*-silenced tobacco. Values are means±SE, n = 30. (C) Daily number of eggs laid by per female whitefly on control empty-vector-inoculated and *rps27A*-silenced tobacco plants. Values are means±SE, n = 30. (D) Relative expression of *RPS27A* gene in RPS27A overexpressing (*OE-RPS27A*) plants. Values are means±SE, n = 8. (E) Survival rate of adult whitefly on control and RPS27A overexpressing (*OE-RPS27A*) tobacco plants. Values are means±SE, n = 30. (F) Daily number of eggs laid by per female whitefly on control and RPS27A overexpressing (*OE-RPS27A*) plants. Values are means±SE, n = 30. (G) Expression of terpene-related genes [*epi-arisotolchene synthase* (*EAS*), *epi-arisotolchene synthase 12* (*EAS12*), *terpenoid cyclase* (*Cyclase*)] in control and *RPS27A*-silencing tobacco plants. Values are means±SE, n = 8. (H) Expression levels of *MYC2* in control and *RPS27A*-silencing tobacco plants. Values are means±SE, n = 8. (I) Effects of whitefly infestation on content of RPS27A protein. (J) & (K) *RPS27A*-silencing and C2 overexpression affect the degradation of JAZ1 protein. Accumulation of JAZ1 protein in control, whiteflies infested, rps27A-silencing or C2 expressing plants was detected by western blot. Same amount of leave samples were collected and JAZ1 protein content was detected by western blot. Asterisks indicate significant differences between different treatments (*P* < 0.05; Student’s t test for all experiments). All experiments were repeated twice with similar results.

To test whether *NtRPS27A* plays a role in tobacco defense against whiteflies, we silenced this gene using VIGS. The silencing efficiency was detected by qRT–PCR analysis (Fig 4A & S4A Fig). We then examined the effects of *NtRPS27A* on the performance of whiteflies. The survival and fecundity of whiteflies were significantly higher on *RPS27A* -silenced plants than those on controls (Fig 4B & 4C). Next, we compared the performance of whiteflies on TYLCV infected and TYLCV infected *RPS27A-VIGS* plants. No marked difference was found on the survival rates of whiteflies between the two treatments (S4B Fig). However, the fecundity of whiteflies on TYLCV infected *RPS27A-VIGS* plants was significantly higher than that on controls (S4C Fig). These results indicate that RPS27A only partially copy the phenotype of TYLCV infection.

Similar to our results, C2 of TYLCSV is reported to suppress JA pathway in *A*. *thaliana* by interacting with CSN5 [47]. However, in our experiments, *NtCSN5*-silenced tobacco plants showed obviously abnormal growth (S5A Fig) and CSN5 barely played a role in plant defense against whiteflies on tobacco plants (S5B–S5D Fig), which may due to the differences of plant species and insects used. To clarify the effects of RPS27A overexpression on whitefly performance, we constructed transgenic tobacco plants with 35S::GFP or 35S::RPS27A-GFP tobacco plants respectively. We screened 10 positive T_0_ individuals for each protein by PCR and qRT-PCR. Bioassay tests showed that overexpression of *NtRPS27A* had no significant effects on survive and fecundity of whiteflies (Fig 4D–4F).

### *RPS27A* affects terpene synthesis by regulating the degradation of JAZ1

To investigate whether NtRPS27A is also involved in plant JA signaling pathway and whitefly defense, we determined the expression of *NtRPS27A* gene in plants after whitefly infestation and JA treatment. Results showed that whitefly infestation and JA treatment did not affect the expression of *RPS27A* (S6A & S6B Fig). To determine how NtRPS27A affects plant defenses, we silenced *RPS27A* and detected the expression levels of genes related to terpene and nicotine synthesis. In line with the results of C2 transgenic plants, transcript levels of *EAS* and *Cyclase* genes were significantly decreased in the *rps27A*-silenced plants compared to VIGS control (Fig 4G). In addition, the transcription of *EAS12* in *rps27A*-silencing plants was also reduced significantly (Fig 4G). However, genes related to nicotine synthesis had no significant difference compared with wild type plants (S6C Fig). These results indicate that TYLCV may suppress plant terpene synthesis by inhibiting RPS27A. The expression of terpene synthesis genes including *EAS*, *EAS12* and *Cyclase* are regulated by MYC2, which is a major transcription factor in JA signaling [42]. Therefore, we examined whether silencing of *NtRPS27A* affects *MYC2* gene expression. Our results showed that silencing of *RPS27A* had no significant effects on the expression of *MYC2* gene (Fig 4H), suggesting that NtRPS27A might regulate MYC2 activity.

MYC2 activity is regulated by JAZ1 protein and degradation of JAZ1 protein by ubiquitination is the switch of MYC2 activation [48,49]. It has been reported that a *N*. *benthamiana* ortholog of NtRPS27A regulates plant growth and the ubiquitin moiety can be cleaved from RPS27A [50]. Thus, we assumed that whitefly infestation may promote the cleavage of RPS27A and degradation of JAZ1 protein. Consistent with our hypothesis, whitefly infestation reduced the amount of NtRPS27A protein in tobacco plants rather than *JAZ1* expression (Fig 4I& S6D), which led us to consider that interaction of C2 with RPS27A or ubiquitin would affect JAZ1 degradation. To test this hypothesis, we first examined whether NtRPS27A silencing and whitefly feeding could regulate JAZ1 degradation. Western blot assay revealed that whitefly infestation led to the degradation of JAZ1 in the control plant (Fig 4J). However, after whitefly infestation, JAZ1 protein had higher accumulation in RPS27A-silenced plants than on control plants (Fig 4J, compare line 2 and 4), demonstrating that NtRPS27A contributed to the degradation of JAZ1. Similarly, JAZ1 protein was more stable in transgenic C2 plants after whitefly feeding (Fig 4K). Taken together, our results suggest that C2 might interact with ubiquitin domain of NtRPS27A to suppress JAZ1 degradation and the activation of JA signaling pathway, and thus promote whitefly performance.

### Interactions of RPS27A with C2 from different begomoviruses

**Fig 5:**
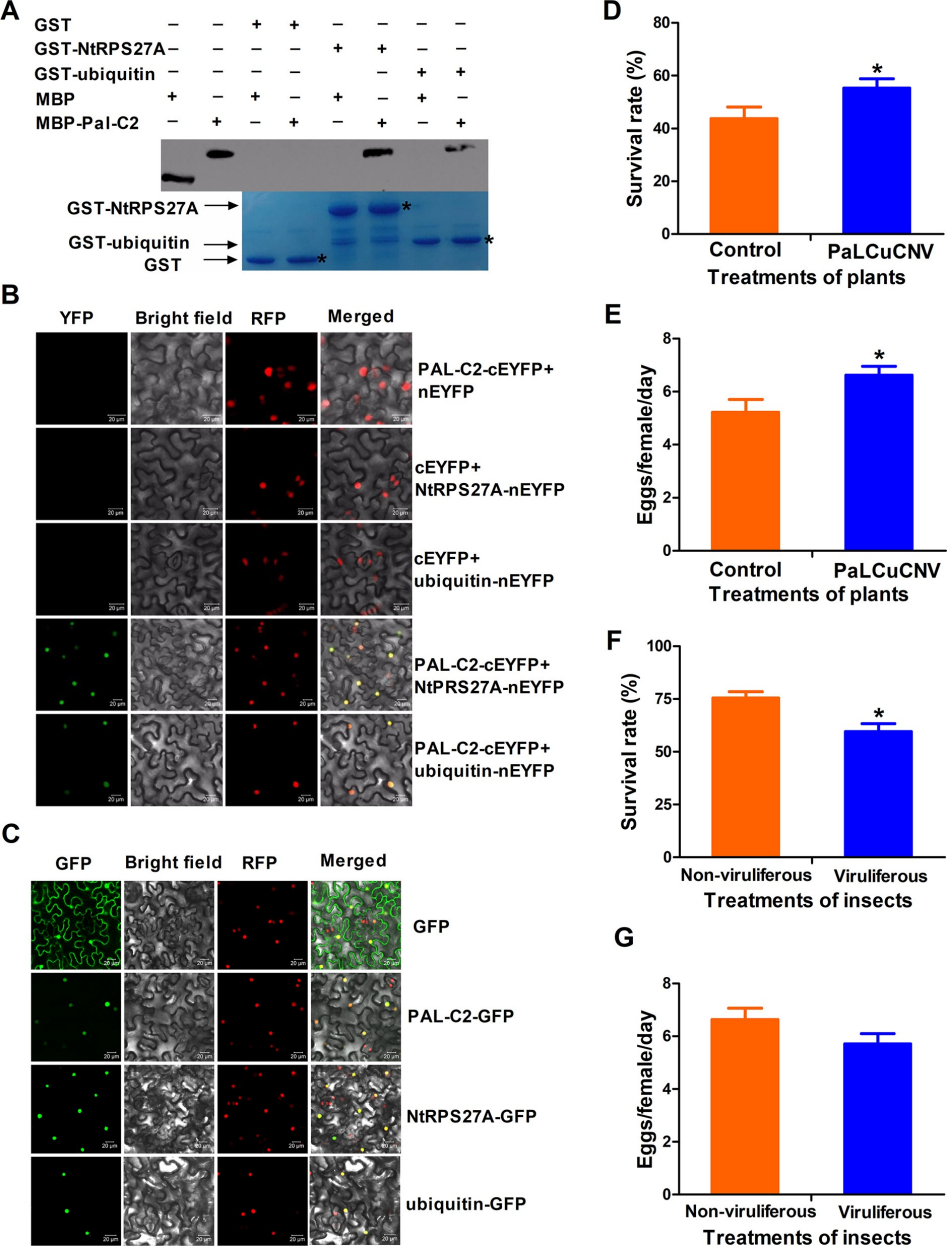
PaLCuCNV C2 interacts with RPS27A and promotes the performance of whitefly. (A) *In vitro* GST pull-down assays. MBP or MBP-PaL-C2 fusion proteins were pull-down by GST or GST-NtRPS27A fusion protein. GST beads were washed and proteins were analyzed by SDS-PAGE western blot. Associated proteins were detected by anti-MBP antibody and gels were stained with Coomassie Brilliant Blue to monitor GST and GST fusion proteins. (B) *In vivo* BiFC analysis of PaL-C2 interaction with NtRPS27A or ubiquitin. Nuclei of tobacco leaf epidermal cells were marked with a RFP fusion protein which is located in nucleus. Bars = 20 mm. (C) Subcellular localization of PaL-C2, NtRPS27A and ubiquitin. Nuclei of tobacco leaf epidermal cells were marked with a RFP fusion protein. Bars = 20 mm. (D) Survival rate of adult whitefly on control and PaLCuCNV-infected tobacco plants. Values are means±SE, n = 30. (E) Daily number of eggs laid by per female whitefly on control empty-vector-inoculated and PaLCuCNV -infected tobacco plants. Values are means±SE, n = 30. (F) Survival rate of non-viruliferous and viruliferous adult whiteflies on cotton plants. Values are means±SE, n = 30. (G) Daily number of eggs laid by per non-viruliferous and viruliferous adult female whiteflies on cotton plants. Values are means±SE, n = 30. Asterisks indicate significant differences between different treatments (*P* < 0.05; Student’s t test for all experiments). All experiments were repeated three times with similar results.

To explore whether interaction with RPS27A is conserved for other begomoviruses C2 proteins, thus promoting vector performance, we performed experiments with papaya leaf curl China virus (PaLCuCNV), another monopartite begomovirus not associated with betasatellite. GST pull-down and BiFC assays showed that PaL-C2 interacted with NtRPS27A by binding to ubiquitin (Fig 5A & 5B). PaL-C2 was also located in nucleus, which is consistent with TYLCV C2 (Fig 5C). We then compared the survival and reproduction of whiteflies on uninfected and PaLCuCNV-infected tobacco plants and found that whitefly survived better and laid more eggs on PaLCuCNV infected plants (Fig 5D & 5E). However, on cotton plants, viruliferous whiteflies had lower survival than non-viruliferous whiteflies, but no difference was observed in fecundity (Fig 5F & 5G).

Next, we studied the evolutionary relationships of C2 proteins from 18 different geminiviruses. As shown in S7 Fig, most of geminiviruses not associated with betasatellites were in the same cluster. Notably, C2 of PaLCuCNV is evolutionally close to TYLCCNV C2. Therefore, we hypothesized that C2 of TYLCCNV might interact with NtRPS27A as well. In contrast with our hypothesis, TYLCCNV C2 did not interact with RPS27A both in yeast and plant (S8 Fig). Sequence alignment of C2 protein from different geminiviruses with or without betasatellites indicated that begomoviruses associated with betasatellites display an amino acid deletion at the 28^th^ amino acid (S9A Fig). To examine whether this amino acid deletion affects C2 and RPS27a interaction, we constructed a mutant TYLCV C2 by deleting the 28^th^ amino acid and a mutant TYLCCNV by inserting the 28^th^ amino acid. However, the mutant TYLCV C2 (SH2-C2_Δ28_) could still interact with RPS27A and the mutant TYLCCNV C2 (TYLCCNV C2_∀28_) still could not (S9B Fig), suggesting that other mechanisms determine the specific interaction between C2 and RPS27A. Previous studies have shown that βC1 protein encoded by TYLCCNB plays a key role in suppressing plant defense against whiteflies as TYLCCNV plus a mutant betasatellite could not promote the performance of whiteflies [27]. These results could indicate that maybe only C2 of monopartite begomoviruses not associated with betasatellites can interact with RPS27A to inhibit plant defense against whiteflies.

### C2- RPS27A interaction is conserved in *Arabidopsis* and tomato

**Fig 6:**
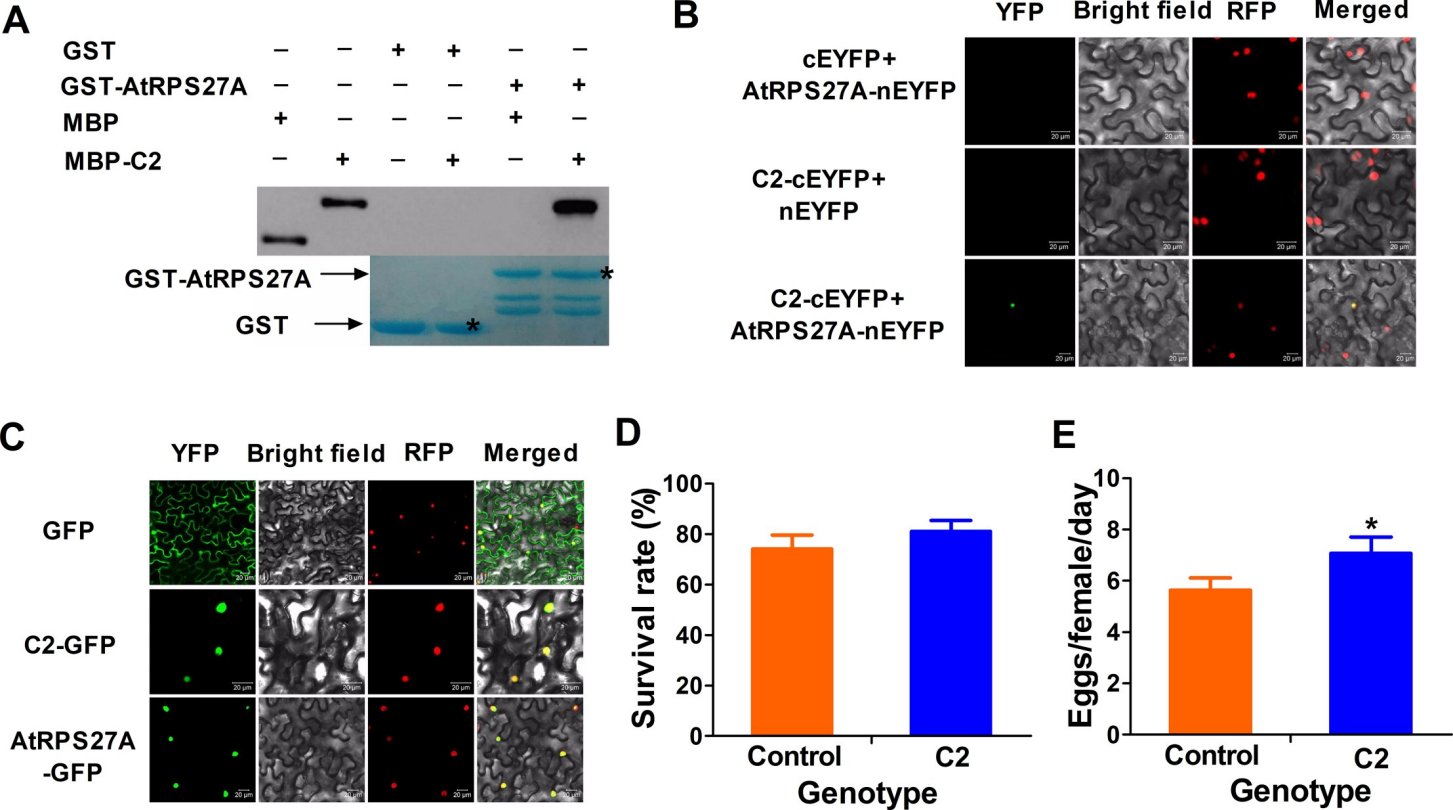
Interaction between TYLCV C2 and AtRPS27A affects whitefly performance. (A) *In vitro* GST pull-down assays. MBP or MBP- C2 fusion proteins were pull-down by GST or GST-AtRPS27A fusion protein. GST beads were washed and proteins were analyzed by SDS-PAGE western blot. Associated proteins were detected by anti-MBP antibody and gels were stained with Coomassie Brilliant Blue to monitor GST and GST fusion proteins. (B) *In vivo* BiFC analysis of C2 interaction with NtRPS27A. Nuclei of tobacco leaf epidermal cells were marked with a RFP fusion protein. Bars = 20 mm. (C) Subcellular localization of C2 and AtRPS27A. Nuclei of tobacco leaf epidermal cells were marked with a RFP fusion protein. Bars = 20 mm. (D) Survival rate of adult whitefly on wild type *Arabidopsis* plants and the transgenic C2 expressing *Arabidopsis* plants. Values are means± SE, n = 30. (E) Daily number of eggs laid by per female whitefly on wild type *Arabidopsis* plants and the transgenic *Arabidopsis* plants expressing C2. Values are means±SE, n = 30. Asterisks indicate significant differences between different treatments (P < 0.05; Student’s t test for all experiments). All experiments were repeated three times with similar results.

Ubiquitin is a highly conserved protein among different species (S10A Fig). Therefore, we asked whether C2 of TYLCV could interact with RPS27A orthologs in *Arabidopsis thaliana* (AtRPS27A). Specific interaction between C2 and AtRPS27A (AT3G62250) was confirmed by BiFC and GST pull-down assays (Fig 6A & 6B). Subcellular location of AtRPS27A was consistent with NtRPS27A (Fig 6C). We further examined if the overexpression of C2 may contribute to the performance of whiteflies on *Arabidopsis*. Results of bioassay tests of whiteflies on wild type *Arabidopsis* plants and the transgenic *Arabidopsis* plants expressing C2 showed significant differences for daily number of eggs laid by per female whitefly, but not for survival rate of adults (Fig 6D & 6E). Taken together, our results suggest that TYLCV C2- RPS27A interaction is well conserved in *Arabidopsis*.

As shown in S10A Fig, ubiquitin domain of tomato RPS27A (SlRPS27A) harbors two amino acid substitutions at 53 and 61 sites. To verify whether these mutations affected the interaction of SlRPS27A with TYLCV C2, we conducted split-luciferase complementation and BIFC assays. Our results demonstrated that the SlRPS27A could also interact with TYLCV C2 protein (S10B & S10C Fig). Taken together, our results suggest that C2-RPS27A interaction is relatively conserved in tobacco, tomato and *Arabidopsis*.

## Discussion

Whiteflies, host plants and geminiviruses have evolved complex relationship [51–53]. When exploring plant-mediated whitefly and geminivirus interaction, one must bear in mind that geminivirus infection may affect the defense of plant hosts, and in turn the behavior and ecology of the whitefly vector. Previously, most attention has been paid to the effects of begomoviruses associated with betasatellites on insect vectors [27,32,33], whereas the effects of begomoviruses in absence of betasatellites on insect vectors and the innermost mechanism have not been stated clearly. Moreover, association of monopartite begomoviruses with betasatellites and bipartite begomoviruses have originated from monopartite begomoviruses by capturing a pathogenic factor [22]. Therefore, elucidation of mechanisms underlying the interactions between monopartite begomoviruses in the absence of betasatellites and their whitefly vectors will help us to explain how sophisticated mutualism has arisen in the geminivirus-insect vector system.

In this study, we found that the infection of tobacco with TYLCV, a monopartite begomovirus in the absence of any satellite, could benefit whiteflies as well (Fig 1). C2 protein encoded by geminiviruses has been described as a transcription factor for viral genes and a suppressor of gene silencing, both post-transcriptional and transcriptional [54–56]. In addition, C2 protein of TYLCSV could regulate plant hormone signaling pathway [38]. Here we demonstrated that TYLCV C2 is also responsible for the suppression of plant defense against whiteflies (Fig 2A & 2B) by interacting with plant RPS27A. RPS27A is a fusion protein consisting of ubiquitin at the N terminus and ribosomal protein S27a at the C terminus (Fig 3A). It can generate free ubiquitin monomer and ribosomal protein S27a after post-transcriptional regulation [50]. Ubiquitin is a highly conserved 8.5 KD protein and exists in all eukaryotes in multiple forms [57–59]. In our study, we found that ubiquitin moiety of RPS27A from different plant species is also highly conserved (S10 Fig). Subcellular localization showed that RPS27A and ubiquitin moiety were located in nucleus. However, the ubiquitin_32-76_ was located in nucleus and cytosol. We assume that some signals on 1–31 amino acids of RPS27A might guide ubiquitin into nucleus.

**Fig 7:**
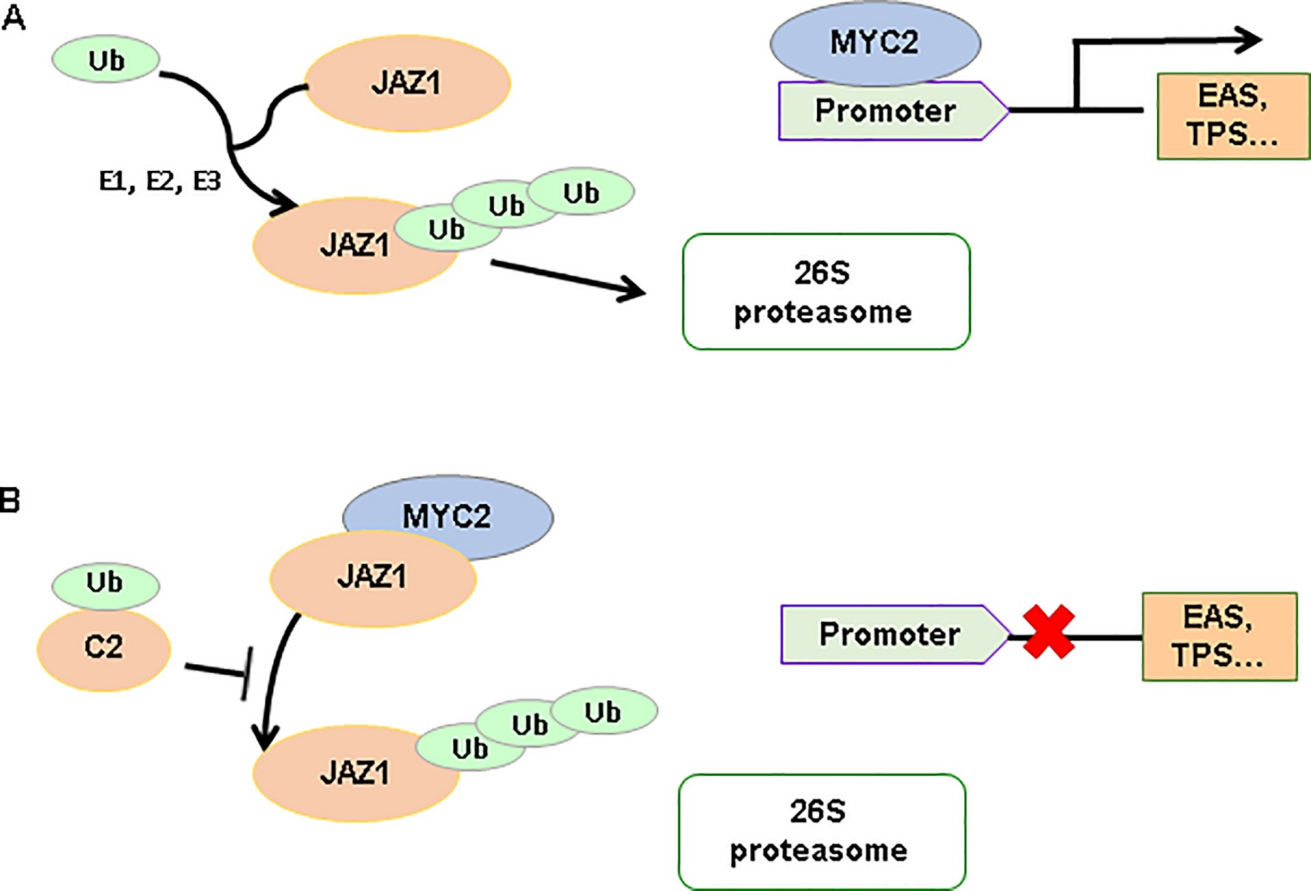
Model for the role of TYLCV C2 in regulating plant defense. (A) When plants are infested by whiteflies, JAZ1 protein can be degraded by ubiquitination via El ubiquitin-activating enzyme (E1), E2 ubiquitin-conjugating enzymes (E2) and E3 ubiquitin–protein ligase (E3) in 26S proteasomes. Then the MYC2 transcription factor is released from JAZ1-MYC2 protein complex, binds to the promoter of defense genes and activates the expression of down-stream defense genes, such as epi-arisotolchene synthase (EAS), and terpene synthase (TPS). As a consequence, plant defense is triggered. (B) When TYLCV and whitefly co-infect plants, TYLCV C2 protein competitively bind to ubiquitin, which lead to the decrease of JAZ1 protein ubiquitination. As a consequence, the MYC2 is bound to JAZ1 and unable to induce the gene expression of down-stream defense genes.

Plant pathogens can manipulate host proteasomes, hijack ubiquitin-mediated degradation, and improve their fitness [60–64]. Our study also showed that TYLCV C2 may interact with ubiquitin moiety of RPS27A *in vivo* and *in vitro* and that the N-terminal ubiquitin of RPS27A is essential for the interaction (Figs 3 & 4). Bioassays of whiteflies on *rps27A* -silenced tobacco plants indicated that RPS27A is a crucial factor for regulation of plant defense (Fig 4A–4C). The JA signaling pathway plays a vital role in plant defense against whitefly and JAZ1 is the on-off switch of MYC2 activity [27,33,49,65]. Binding of MYC2 with JAZ1 inhibits the activation of MYC2, thus affecting the transcription of resistance genes in JA signaling pathway [33]. Hence, degradation of JAZ1 protein would activate the defense of plants. In our study, silencing of *rps27A* inhibited JAZ1 degradation and expression of genes related to terpene synthesis (Fig 4G, 4J and 4K), which is consistent with the overexpression of C2. Taken together, RPS27A might regulate plant defense by manipulating the degradation of JAZ1 protein in the JA signaling pathway and virus C2 circumvents this process by inhibiting the function of RPS27A/ubiquitin (Fig 7).

Previous results demonstrated that the betasatellite associated with the monopartite begomovirus TYLCCNV is important for plant mediated virus and vector interaction as TYLCCNV with mutant TYLCCNB could not promote the performance of whiteflies [27]. Interaction between TYLCCNB and plant MYC2 leads to better performance of whitefly [33]. Our results showed that, contrary to monopartite begomoviruses without betasatellite (TYLCV and PalCuCNV), C2 of TYLCCNV did not interact with RPS27A (S8 Fig), suggesting that only C2 of monopartite begomoviruses without betasatellites might have acquired the function to interact with RPS27A and thus suppress plant JA signaling pathway. Taken together, these results indicate that begomoviruses associated with and not associated with betasatellites utilize different mechanisms to suppress plant JA defense against insects.

In the field, a plant can be infected by various viruses and one virus may have a broad range of host plants species. Given ubiquitin is highly conserved, it seems reasonable to postulate that such an interaction between RPS27A and begomoviruses may occur in many plant-virus systems. In our experiments, we found that TYLCV C2 may also interact with *Arabidopsis* RPS27A and that the expression of TYLCV C2 in *Arabidopsis* could increase the suitability of plants for whiteflies (Fig 6). Similar to our results, C2 of TYLCSV is reported to interrupt ubiquitination in *A*. *thaliana* by interaction with COP9 signalosome complex subunit CSN5 [47]. Tomato CSN5 has been reported to be associated with tomato defense against herbivorous *M*. *sexta* larvae and the necrotrophic fungal pathogen *Botrytis cinereal* by affecting JA and SA content in plants [66]. However, in our experiments, CSN5 barely played a role in plant defense against whiteflies in tobacco plants (S5B–S5D Fig), which may due to the differences of insects used in these studies. In addition, the phenotype of CSN5-silencing tobacco and tomato plants are rather different (S5A Fig), suggesting that CSN5 may have different roles in tobacco and tomato. Previous studies have shown that betasatellites of begomoviruses could also subvert plant ubiquitination. βC1 of cotton leaf curl Multan betasatellite regulates the plant ubiquitination pathway for effective infection by interaction with NbSKP1 [67]. Interaction of βC1 of TYLCCNB with NtRFP1 attenuates disease symptoms [68]. It appears that although the detailed mechanisms are different, hijacking the plant ubiquitination process might be a common strategy used by begomoviruses to regulate plant defenses. Taken together, we deem that suppression of RPS27A by TYLCV C2, leading to decreased plant defense against whitefly, might be conserved among different combinations of TYLCV and host plants.

In summary, we elaborated how begomoviruses not associated with betasatellites manipulate plant defense. We found that infection of monopartite begomoviruses either TYLCV or PaLCuCNV promoted the performance of whitefly vectors. C2 of these begomoviruses directly interacted with plant ubiquitin to compromise the activation of terpene synthase genes, thereby reducing plant resistance to whiteflies (Fig 7). Function of C2 may be well conserved in monopartite begomoviruses not associated with betasatellites. Although some monopartite begomoviruses such as TYLCCNV have evolved a different strategy to interfere with plant defenses, our data, combined with some related studies in the literature, indicate that regulation of ubiquitination progress may be an evolutionarily conserved strategy of begomovirus to suppress plant defense. Our findings also explain how sophisticated mutualism has evolved in the begomoviruses insect vector system and might lead to new strategies to combat their spread.

## Materials and methods

### Plants, viruses and whiteflies

Infectious clones of TYLCV (SH2, GenBank accession no. AM282874), TYLCCNV (AJ319675.1) TYLCCNB (AJ781300) and PaLCuCNV (FN256260) were described previously [69–71]. Tobacco (*Nicotiana tabacum* cv. NC89; *N*. *benthamiana* line H2B-RFP) plants were cultivated in a greenhouse under natural lighting and controlled temperature at 25 ± 3°C. To obtain virus-infected tobacco, the leaf of each plant at the two-to-three true leaf stage was inoculated by agroinoculation as previously described and cultured to the six-to-seven true-leaf stage. Virus infection of plants was confirmed by PCR. The control tobacco plants were inoculated with empty vector pBIN-plus. Non-viruliferous *B*. *tabaci* MEAM1 [mitochondrial cytochrome oxidase subunit I (mt*COI*) GenBank Accession no. GQ332577] were maintained on tobacco or cotton plants in an insectary at 25 ± 1°C, 70 ± 10% relative humidity and a 14/10 h light/dark cycle.

### Assessment of plant suitability via whitefly performance

Newly emerged (2 days old) whiteflies (5 males and 5 females) were collected and released into each of three clip cages secured to the abaxial surface of a tobacco plant leaf (third to fifth leaves from the top). Ten plants were used in each treatment, thus there were 30 clip cages per treatment and each is a replicate. All experiments were repeated three times. Seven days after infestation, adult survival and number of eggs laid by whiteflies were recorded to assess host plant suitability.

### Direct effects of begomovirus infection on whitefly performance

Newly emerged whiteflies (1000 individuals) from cotton plants were transferred to uninfected and TYLCV or PaLCuCNV–infected tomato plants respectively, which had been inoculated with begomovirus for 25 d. Two days later,10 non-viruliferous whiteflies (5 females and 5 males) and 10 viruliferous whiteflies (5 females and 5 males) were collected and released into separate clip cages respectively, fixed to symmetrical side of the same cotton plant leaf. The performance of whiteflies on cotton plants was determined as above.

### Construction of transgenic plants

To generate TYLCV C2, C4, V2 and RPS27A-GFP transgenic tobacco plants, C2, C4, V2 and RPS27A were amplified and cloned into modified binary vector pCAMBIA1300 or pCAMBIA1305 with GFP to obtain overexpression vectors. The expression vectors were sequenced to confirm fidelity. Transgenic plants were generated by *Agrobacterium* mediated transformation, and positive plants were determined by PCR analysis. Wild type plants were used as control of C2, V2 and C4 transgenic tobacco plants, and transgenic plants expressing pCAMBIA1305-GFP were used as controls for RPS27A-GFP plants.

### Yeast two-hybrid analysis

A partial sequence of C2, named C2_1-78_, lacking 59 amino acids of the C terminus was cloned into pGBKT7 vector and RPS27A was cloned into pGADT7 vector (Clontech). Assessment of the interaction between C2 and RPS27A was performed according to the manufacturer’s protocol (Clontech) and plated on SD-Leu-Trp-His-Ade plates with 2 mM 3-amino-1, 2, 4-triazole and X-α-Gal. The reagents used in yeast two-hybrid analysis were purchased from Clontech Laboratories, and the procedures of the manufacturer’s protocol were followed.

### Pull-down Assay

The recombinant GST and MBP tag proteins were purified using GST- (GE Healthcare) or MBP-Trap (New England Biolabs) according to the manufacturer’s instructions. The pulled-down proteins were separated on 12% SDS-PAGE gels and detected by western blot using anti-MBP antibody (Abcam). The GST and GST fusion proteins were detected by Coomassie Brilliant Blue stain.

### BIFC and split-luciferase complementation assay

C2 of TYLCV, TYLCCNV or PaLCuCNV and TYLCCNB were fused to the C-terminal domain of YFP. The segments of RPS27A were fused to the N-terminal domain of YFP to generate ubiquitin_32-76_- nYFP, ubiquitin-nYFP, AtRPS27A- nYFP and NtRPS27A-nYFP. Different combinations of the *A*. *tumefaciens* clones expressing the fusion proteins were co-infiltrated into the leaves of 3-week-old *N*. *benthamiana* line H2B plants, which can express RFP fusion protein located in the nucleus. Two days after incubation, RFP fluorescence and YFP fluorescence were imaged with a Zeiss LSM710 confocal microscope. For split luciferase complementation (SLC) assay, C2 and indicated RPS27A-related protein were constructed into pCAMBIA-GW-nLUC and pCAMBIA-GW-cLUC plasmids, transiently expressed in *N*. *benthamiana*. After sprayed with 1 mM beetle luciferin (Promega), the signal was captured using a Photek camera (HRPCS5; Photek) for 10 min.

### Subcellular localization assay

For the subcellular localization study, TYLCV C2 and RPS27A were inserted into a modified pCAMBIA -1305 contained GFP, and transformed into *A*. *tumefaciens*. The cultures were infiltrated into *N*. *benthamiana* line H2B plants. Forty-eight hours after infiltration, the leaves were imaged with a Zeiss LSM710 confocal microscope.

### Virus-induced gene silencing (VIGS) assays

A fragment (300-500bp) of each targeted gene was amplified from tobacco leaf cDNA using a gene-specific primer pair (S1 Table) and cloned into pBIN2mDNA1 plasmid to generate the gene-silencing vectors [72]. Then we transformed the vectors into *A*. *tumefaciens* strain EHA105 by electroporation. The method for VIGS was as described previously [72]. All plants were grown at the same conditions as described above. At six-to-seven true-leaf stage, total RNA was isolated 3 days before bioassay. Silencing efficiency was determined by qRT-PCR (S1 Table).

### Quantitative real-time PCR (qRT-PCR)

For whitefly infestation, approximately 1000 adult whiteflies from uninfected tobacco plants were released and allowed to infest one empty-vector-inoculated plant in a cage or one virus-infected plant in another cage. After 72 h of feeding, the adults were discarded. Total RNA of empty-vector inoculated, whitefly-infested, TYLCV-infected and co-infection plants were extracted with Trizol^TM^ and cDNA was synthesized using the SYBR PrimeScriptRT-PCR Kit II (Takara, Dalian, China). qRT-PCRs were performed using the BIO-RAD CFX96 PCR System (Bio-Rad, California, USA). Each gene was analyzed in triplicate technical repeats for each of the eight or six biological replicates. The average threshold cycle (Ct) was calculated per sample. After normalized to *GAPDH*, relative expression levels of genes were calculated with the 2^-ΔΔCT^method.

### Quantitation of protein content

For whitefly infestation, approximately 1000 adult whiteflies from uninfected tobacco plants were released and allowed to infest one empty-vector-inoculated, RPS27A silenced, wild type or C2 expressing plant, and each plant was put into one cage. After 72 h of feeding, leaves from the plants in each of the four treatments were harvested. The content of JAZ1 protein was performed by JAZ1 antibody, which is a polyclonal antibody (Huaan Company, China). Polyclonal antibodies were produced by immunizing rabbit with the prokaryotic protein JAZ1-GST.

### Statistical analysis

Statistical significance was evaluated using one-way ANOVA at a 0.05 level followed by LSD tests with nested design for gene expression. Whitefly performance experiments were analyzed using t test at 0.05 levels. Data in percentages (adult survival) were transformed by arcsine square root before analysis. All data analyses were conducted using the software SPSS19.0.

## Supporting information

S1 Fig JA signaling pathway is associated with plant defense. (A) Relative expression of *Cyclase* gene in *cyclase* -silenced tobacco plants. Values are means ±SE, n = 8. (B) Survival rate of adult whitefly on control empty-vector-inoculated and *cyclase* -silenced tobacco plants. Values are means±SE, n = 30. (C) Daily number of eggs laid by per female whitefly on control empty-vector-inoculated and *cyclase*-silenced tobacco plants. Values are means±SE, n = 30. (D) Relative expression of *MYC2* gene in *myc2* -silenced tobacco plants. Values are means ±SE, n = 8. (E) Survival rate of adult whitefly on control empty-vector-inoculated and *myc2* -silenced tobacco plants. Values are means±SE, n = 30. (F) Daily number of eggs laid by per female whitefly on control empty-vector-inoculated and *myc2* -silenced tobacco plants. Values are means±SE, n = 30. (G) Expression of *ODC* in control and *odc*-silenced plants. Values are means±SE, n = 8. (H) Survival rate of adult whitefly on control empty-vector-inoculated and *ODC*-silenced tobacco plants. Values are means±SE, n = 30. (I) Daily number of eggs laid by per female whitefly on control empty-vector-inoculated and *odc*-silenced tobacco plants. Values are means±SE, n = 30. Asterisks indicate significant differences between different treatments (P < 0.05; Student’s t test for all experiments). All experiments were repeated three times with similar results.
(TIF)

S2 Fig TYLCV V2 and C4 are not necessary for inhibiting plant defense against whitefly. (A) Phenotype of wild type tobacco plants (right) and transgenic tobacco plants expressing C4 (left). (B) Survival rate of whitefly on wild type tobacco plants and transgenic tobacco plants expressing TYLCV V2. Values are means ± SE, n = 30. (C) Daily number of eggs laid by per female whitefly on wild type tobacco plants and transgenic tobacco plants expressing V2. Values are means ± SE, n = 30. (D) Survival rate of adult whitefly on wild type tobacco plants and the transgenic tobacco plants expressing C4. Values are means±SE, n = 30. (E) Daily number of eggs laid per female whitefly on wild type tobacco plants and C4 expressing tobacco plants. Values are means±SE, n = 30. Asterisks indicate significant differences between different treatments (P < 0.05; Student’s t test for all experiments). All experiments were repeated three times with similar results.
(TIF)

S3 Fig C-terminal part of NtRPS27A (NtRPS27Ac) did not interact with TYLCV C2 protein. (A) Interaction of RPS27Ac and TYLCV C2 was detected by BIFC. Nuclei of tobacco leaf epidermal cells were marked with a RFP fusion protein that is located in Nuclei. Bars = 20 mm. (B) *In vitro* GST pull-down assays. MBP or MBP-SH2-C2 fusion proteins were pull-down by GST or GST-NtRPS27Ac fusion proteins. GST beads were washed and proteins were analyzed by SDS-PAGE western blot. Associated proteins were detected by anti-MBP antibody and gels were stained with Coomassie Brilliant Blue to monitor GST and GST fusion proteins.
(TIF)

S4 Fig RPS27A plays partial role in tobacco-TYLCV-whitefly interaction. (A) Relative expression of *RPS27A* gene in plants. Values are means±SE, n = 12. (B) Survival rate of adult whiteflies on RPS27A-VIGS and TYLCV infected RPS27A-VIGS tobacco plants. Values are means±SE, n_RPS27A-VIGS_ = 29, n_TYLCV+RPS27A-VIGS_ = 30_._ (C) Daily number of eggs laid by per female whitefly on RPS27A-VIGS plants and TYLCV infected RPS27A-VIGS tobacco plants. Values are means±SE, n_RPS27A-VIGS_ = 29, n_TYLCV+RPS27A-VIGS_ = 30. Asterisks indicate significant differences between different treatments (P < 0.05; Student’s t test for all experiments). All experiments were repeated two times with similar results.
(TIF)

S5 Fig Silencing of CSN5 did not affect whitefly performance. (A) Growth phenotype of *csn5* silencing tobacco plants. (B) Relative expression of *CSN5* gene in *csn5*-silencing tobacco plants. Values are means±SE, n = 8. (C) Survival rate of adult whiteflies on control empty-vector-inoculated and *csn5-silencing* tobacco plants. Values are means±SE, n = 26. (D) Daily number of eggs laid by per female whitefly on control empty-vector-inoculated and *csn5-silencing* tobacco plants. Values are means±SE, n = 26. Asterisks indicate significant differences between different treatments (P < 0.05; Student’s t test for all experiments). All experiments were repeated two times with similar results.
(TIF)

S6 Fig RPS27A expression and effects of silencing rps27A on JA signaling. (A) Effects of whitefly infestation on the expression of *RPS27A* gene. Values are means±SE, n = 8. (B) Effects of JA treatment on the expression of *RPS27A* gene. Values are means±SE, n = 8. (C) Expression of nicotine-related genes *ADC1*, *QPT* and *ODC* in control and RPS27A-silencing tobacco plants. Values are means±SE, n = 8. (D) *JAZ1* expression in plants with different treatments. Values are means±SE, n = 8. All experiments were repeated three times with similar results.
(TIF)

S7 Fig Phylogenetic analysis of C2 from different begomoviruses. The phylogenetic analysis was conducted with MEGA5. Neighbor-Joining method and a bootstrap analysis of 1000 replicates were used. Bootstrap values were shown in the cladogram. ☆, bipartite virus; ▲, monopartite virus lacking of satellites; ●, monopartite virus with satellites; *, virus used in this study; □, WDV, Wheat dwarf virus (a mastrevirus).
(TIF)

S8 Fig C2 of TYLCCNV Y10 could not interact with NtRPS27A protein. (A) Interaction between TYLCCNV-C2 and NtRPS27A in the yeast two-hybrid system. Yeast strain Y2H Gold co-transformed with the indicated plasmids was spotted on synthetic medium SD-Leu-Trp-His with x-α-gal and 2 mM 3-amino-1,2,4-triazole. The empty vectors pGBKT7 and pGADT7 were used as negative controls. (B) *In vivo* BiFC analysis of TYLCCNV-C2 interaction with NtRPS27A. No fluorescence signal was observed suggesting that TYLCCNV-C2 and NtRPS27A did not interact. Nuclei of tobacco leaf epidermal cells were marked with a RFP fusion protein. Bars = 50 mm.
(TIF)

S9 Fig Sequence alignments of C2 protein and function of 28th amino acid. (A) Sequence alignment of C2 among different begomoviruses. The No. 1 virus is a bipartite virus; 2 to 9 are monopartite viruses not associated with satellites; 10 to 15 are monopartite viruses with satellites. (B) The 28^th^ amino acid of C2 protein did not determine the interaction with NtRPS27A. TYLCCNV-C2_∀28_ indicates insertion of 28^th^ amino acid into C2 protein of TYLCCNV; SH2-C2_Δ28_ indicates TYLCV SH2 C2 protein which has a 28^th^ amino acid deletion.
(TIF)

S10 Fig Interaction between TYLCV C2 and SlRPS27A. (A) Sequence alignments of ubiquitin moiety of RPS27A from different plants. (B) *In vivo* split-luciferase complementation assay of C2 interaction with SlRPS27A. (C) *In vivo* BiFC assay of C2 interaction with SlRPS27A. Nuclei of tobacco leaf epidermal cells were marked with a RFP fusion protein H2B-RFP. Bars = 20 mm or 50 mm.
(TIF)

S1 Table Primers used in this article. (DOCX)
