# Supplementary figures and images for: Plant begomoviruses subvert ubiquitination to suppress plant defenses against insect vectors
Source: PLoS Pathog. 2019 Feb 21;15(2):e1007607. doi: 10.1371/journal.ppat.1007607 (PMC6400417; doi:10.1371/journal.ppat.1007607)

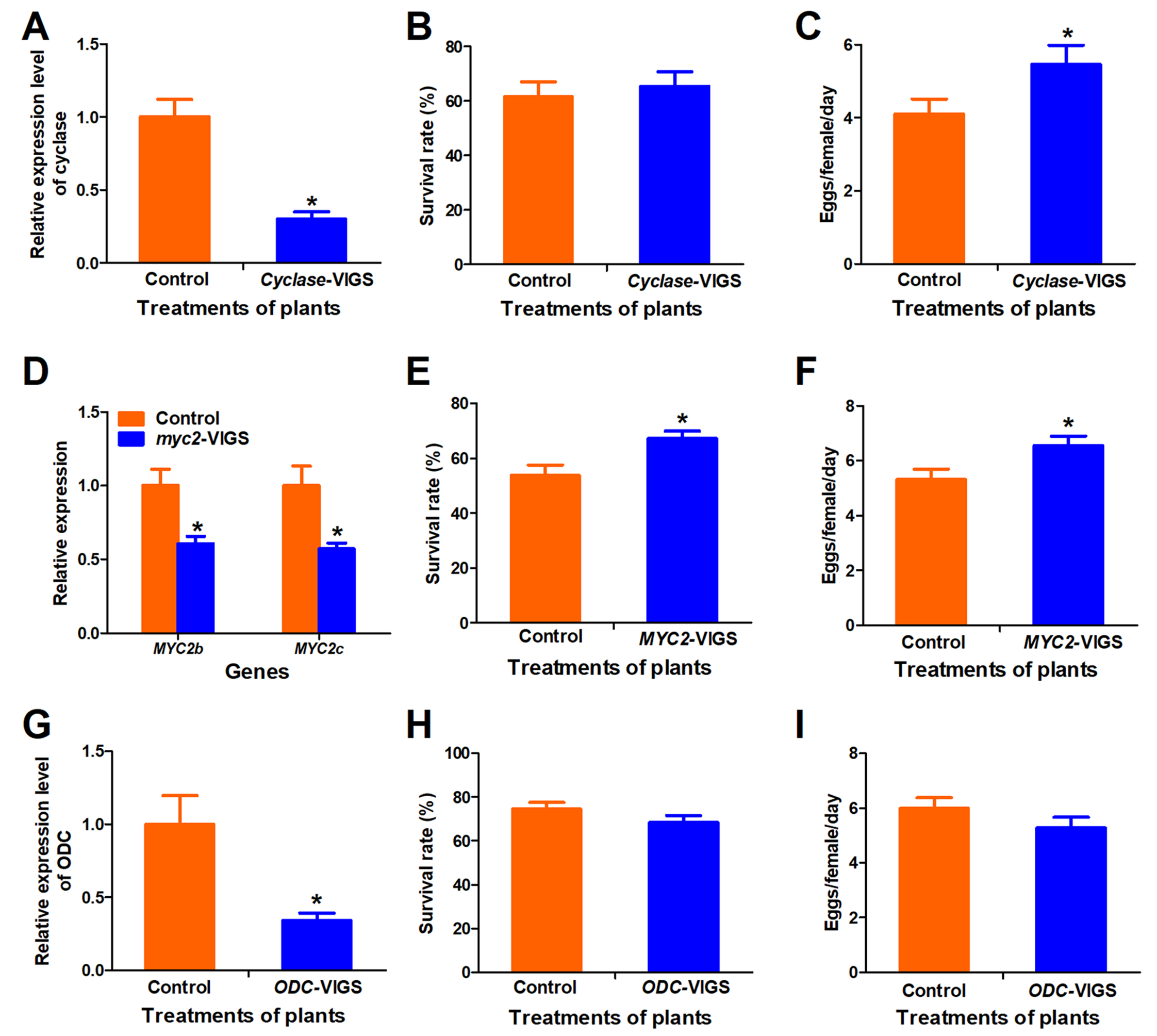

Supplement: S1 Fig — (A) Relative expression of Cyclase gene in cyclase -silenced tobacco plants. Values are means ±SE, n = 8. (B) Survival rate of adult whitefly on control empty-vector-inoculated and cyclase -silenced tobacco plants. Values are means±SE, n = 30. (C) Daily number of eggs laid by per female whitefly on control empty-vector-inoculated and cyclase-silenced tobacco plants. Values are means±SE, n = 30. (D) Relative expression of MYC2 gene in myc2 -silenced tobacco plants. Values are means ±SE, n = 8. (E) Survival rate of adult whitefly on control empty-vector-inoculated and myc2 -silenced tobacco plants. Values are means±SE, n = 30. (F) Daily number of eggs laid by per female whitefly on control empty-vector-inoculated and myc2 -silenced tobacco plants. Values are means±SE, n = 30. (G) Expression of ODC in control and odc-silenced plants. Values are means±SE, n = 8. (H) Survival rate of adult whitefly on control empty-vector-inoculated and ODC-silenced tobacco plants. Values are means±SE, n = 30. (I) Daily number of eggs laid by per female whitefly on control empty-vector-inoculated and odc-silenced tobacco plants. Values are means±SE, n = 30. Asterisks indicate significant differences between different treatments (P < 0.05; Student’s t test for all experiments). All experiments were repeated three times with similar results. (TIF) [file ppat.1007607.s001.tif]

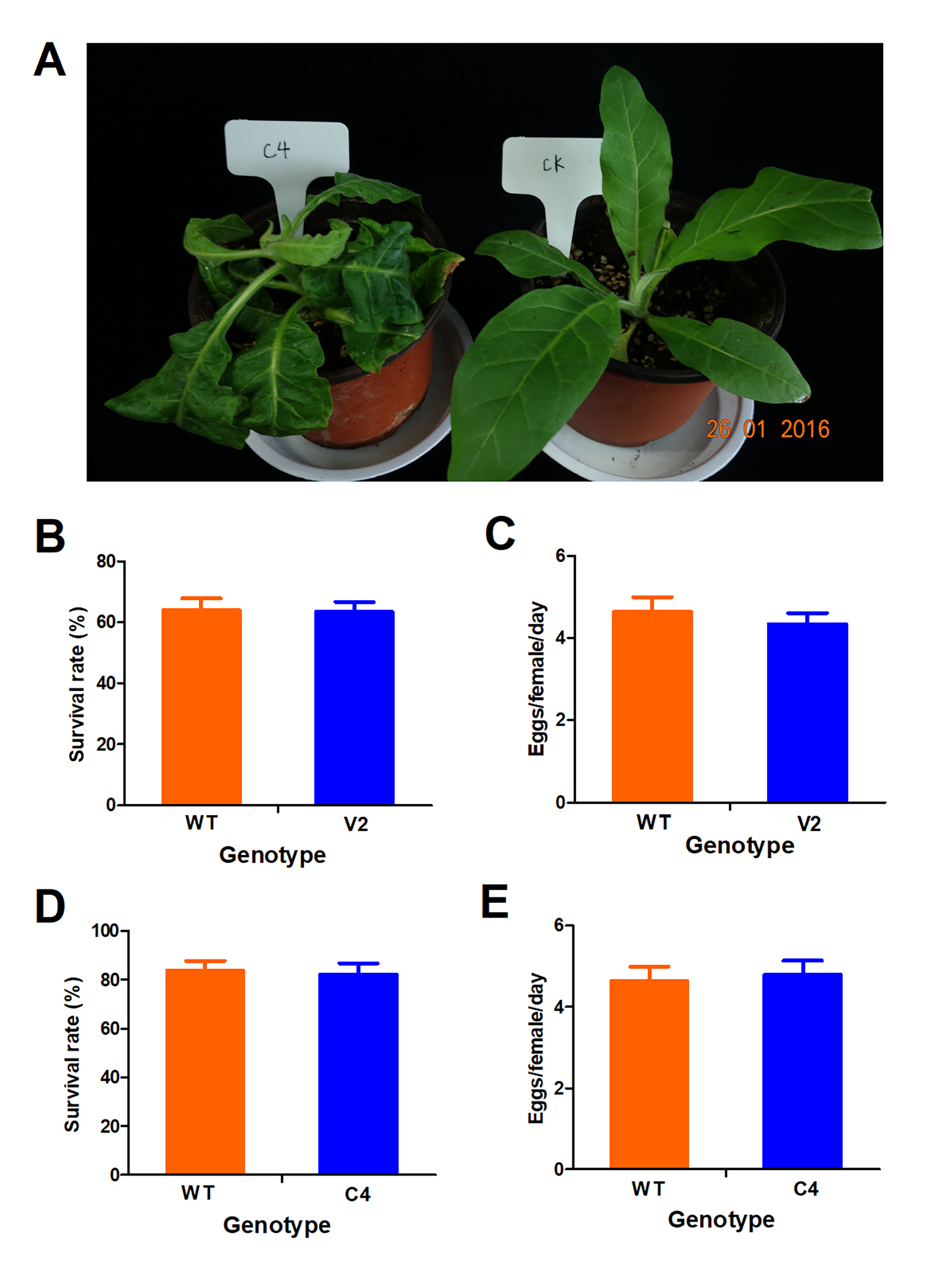

Supplement: S2 Fig — (A) Phenotype of wild type tobacco plants (right) and transgenic tobacco plants expressing C4 (left). (B) Survival rate of whitefly on wild type tobacco plants and transgenic tobacco plants expressing TYLCV V2. Values are means ± SE, n = 30. (C) Daily number of eggs laid by per female whitefly on wild type tobacco plants and transgenic tobacco plants expressing V2. Values are means ± SE, n = 30. (D) Survival rate of adult whitefly on wild type tobacco plants and the transgenic tobacco plants expressing C4. Values are means±SE, n = 30. (E) Daily number of eggs laid per female whitefly on wild type tobacco plants and C4 expressing tobacco plants. Values are means±SE, n = 30. Asterisks indicate significant differences between different treatments (P < 0.05; Student’s t test for all experiments). All experiments were repeated three times with similar results. (TIF) [file ppat.1007607.s002.tif]

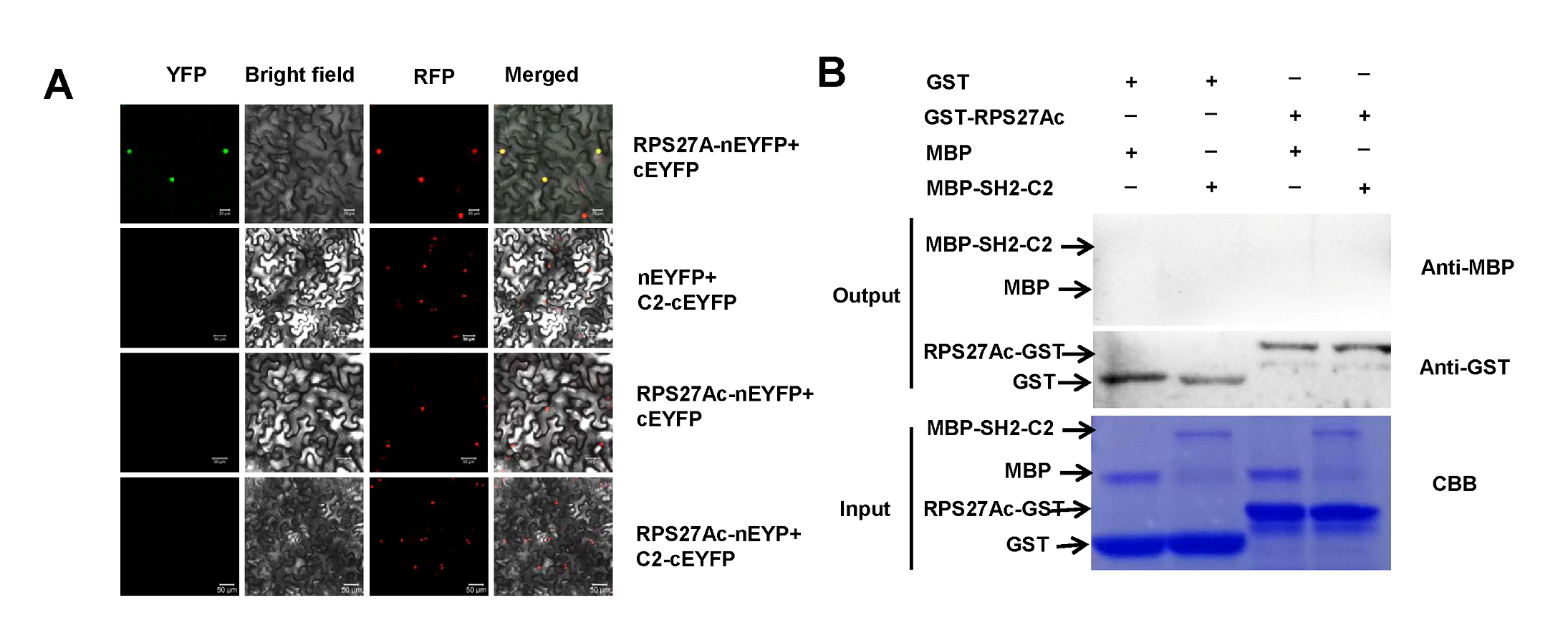

Supplement: S3 Fig — (A) Interaction of RPS27Ac and TYLCV C2 was detected by BIFC. Nuclei of tobacco leaf epidermal cells were marked with a RFP fusion protein that is located in Nuclei. Bars = 20 mm. (B) In vitro GST pull-down assays. MBP or MBP-SH2-C2 fusion proteins were pull-down by GST or GST-NtRPS27Ac fusion proteins. GST beads were washed and proteins were analyzed by SDS-PAGE western blot. Associated proteins were detected by anti-MBP antibody and gels were stained with Coomassie Brilliant Blue to monitor GST and GST fusion proteins. (TIF) [file ppat.1007607.s003.tif]

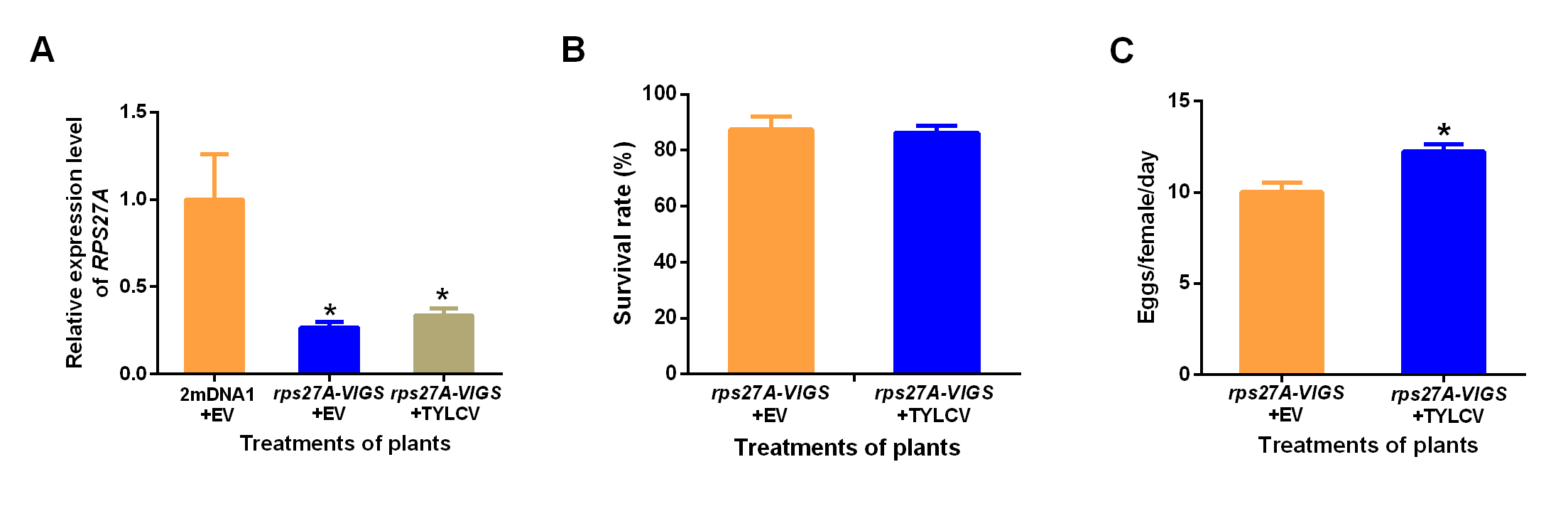

Supplement: S4 Fig — (A) Relative expression of RPS27A gene in plants. Values are means±SE, n = 12. (B) Survival rate of adult whiteflies on RPS27A-VIGS and TYLCV infected RPS27A-VIGS tobacco plants. Values are means±SE, nRPS27A-VIGS = 29, nTYLCV+RPS27A-VIGS = 30. (C) Daily number of eggs laid by per female whitefly on RPS27A-VIGS plants and TYLCV infected RPS27A-VIGS tobacco plants. Values are means±SE, nRPS27A-VIGS = 29, nTYLCV+RPS27A-VIGS = 30. Asterisks indicate significant differences between different treatments (P < 0.05; Student’s t test for all experiments). All experiments were repeated two times with similar results. (TIF) [file ppat.1007607.s004.tif]

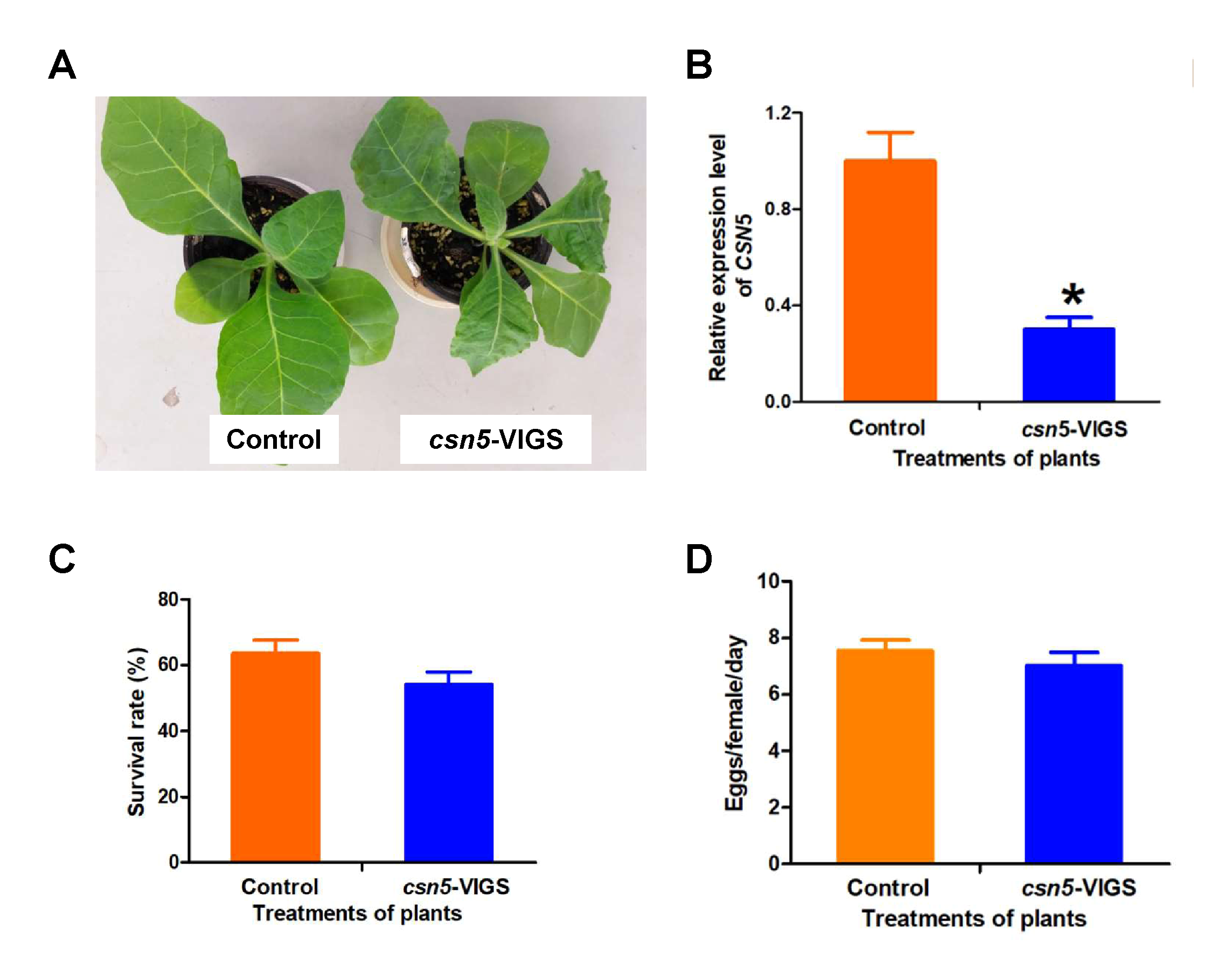

Supplement: S5 Fig — (A) Growth phenotype of csn5 silencing tobacco plants. (B) Relative expression of CSN5 gene in csn5-silencing tobacco plants. Values are means±SE, n = 8. (C) Survival rate of adult whiteflies on control empty-vector-inoculated and csn5-silencing tobacco plants. Values are means±SE, n = 26. (D) Daily number of eggs laid by per female whitefly on control empty-vector-inoculated and csn5-silencing tobacco plants. Values are means±SE, n = 26. Asterisks indicate significant differences between different treatments (P < 0.05; Student’s t test for all experiments). All experiments were repeated two times with similar results. (TIF) [file ppat.1007607.s005.tif]

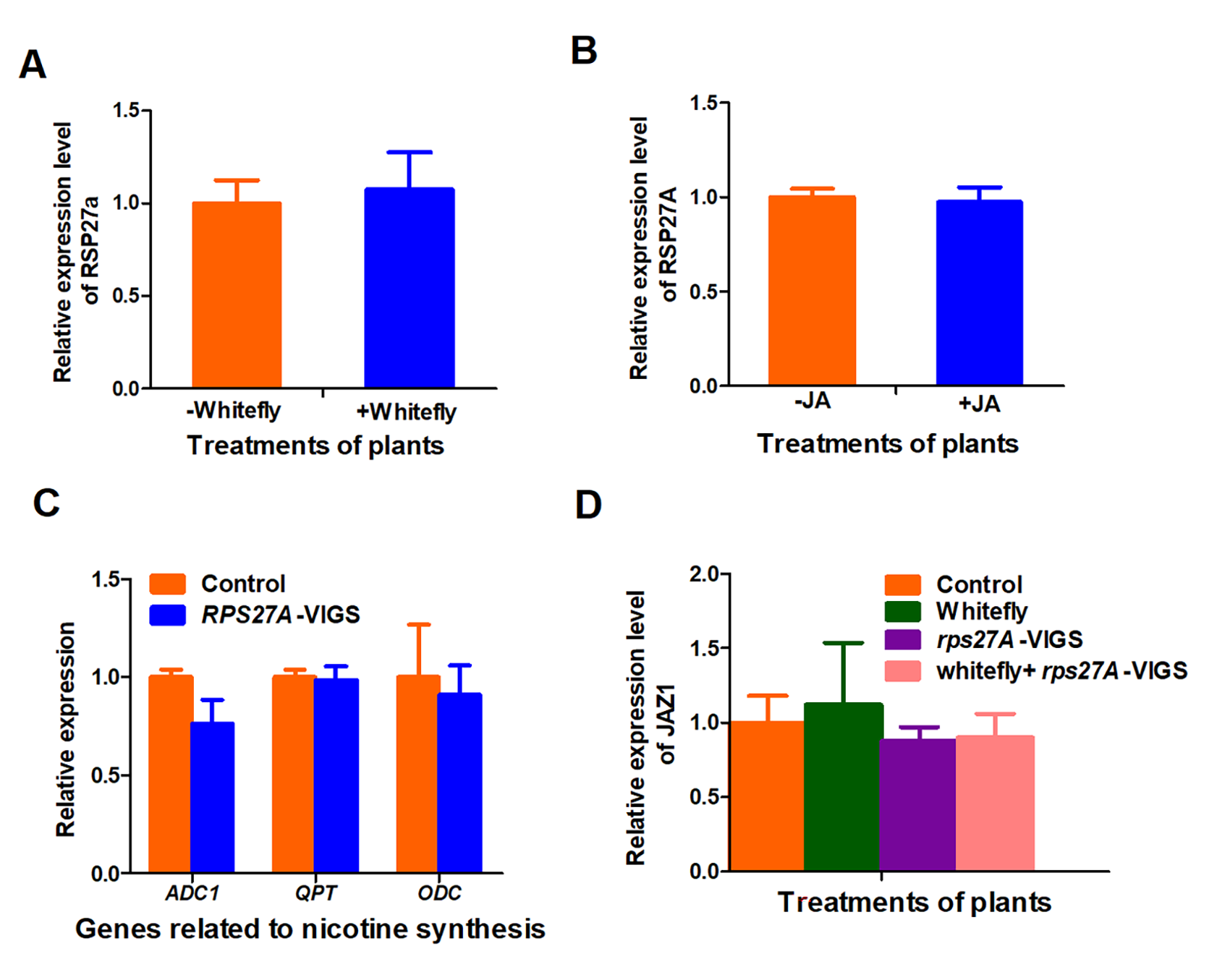

Supplement: S6 Fig — (A) Effects of whitefly infestation on the expression of RPS27A gene. Values are means±SE, n = 8. (B) Effects of JA treatment on the expression of RPS27A gene. Values are means±SE, n = 8. (C) Expression of nicotine-related genes ADC1, QPT and ODC in control and RPS27A-silencing tobacco plants. Values are means±SE, n = 8. (D) JAZ1 expression in plants with different treatments. Values are means±SE, n = 8. All experiments were repeated three times with similar results. (TIF) [file ppat.1007607.s006.tif]

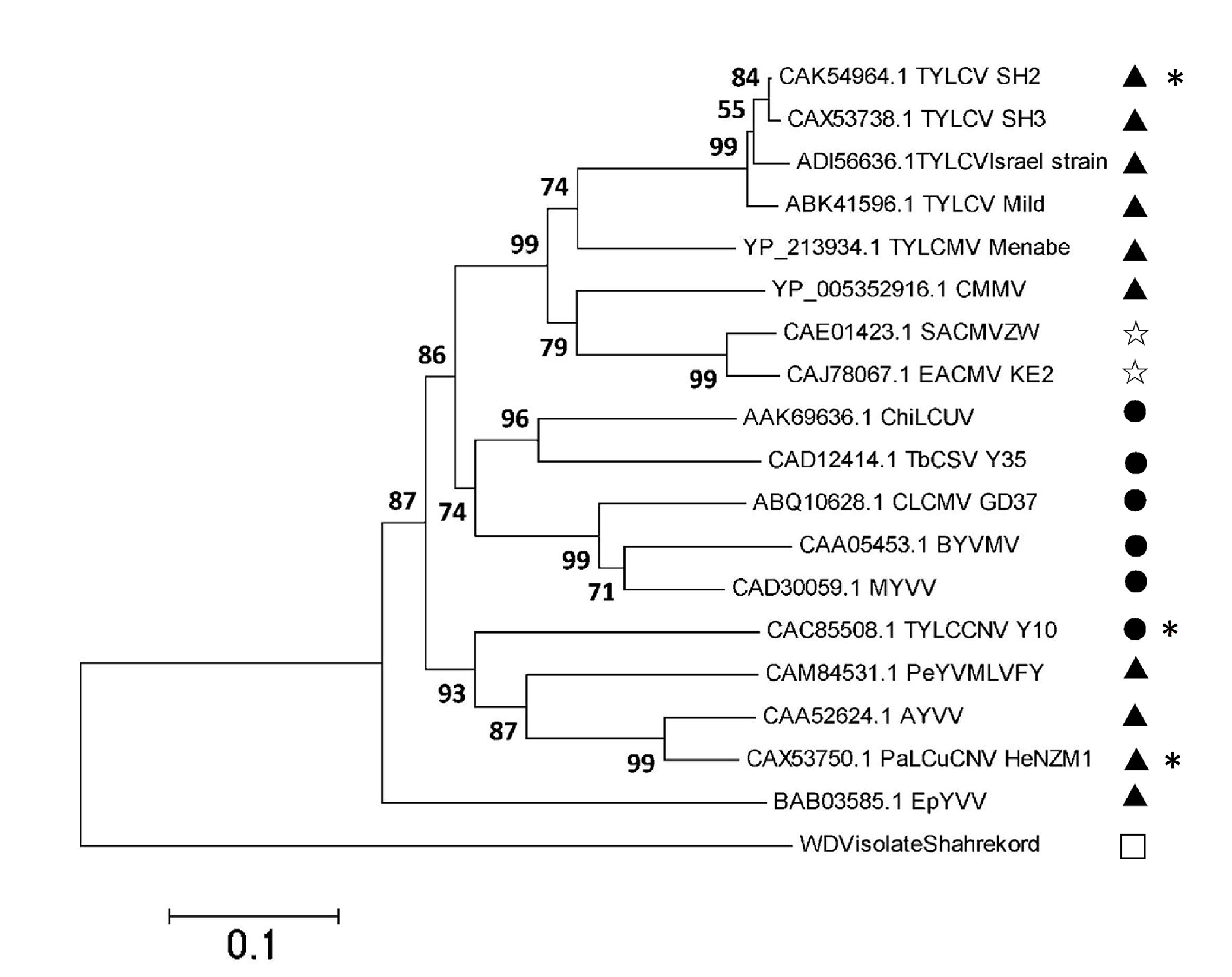

Supplement: S7 Fig — The phylogenetic analysis was conducted with MEGA5. Neighbor-Joining method and a bootstrap analysis of 1000 replicates were used. Bootstrap values were shown in the cladogram. ☆, bipartite virus; ▲, monopartite virus lacking of satellites; ●, monopartite virus with satellites; *, virus used in this study; □, WDV, Wheat dwarf virus (a mastrevirus). (TIF) [file ppat.1007607.s007.tif]

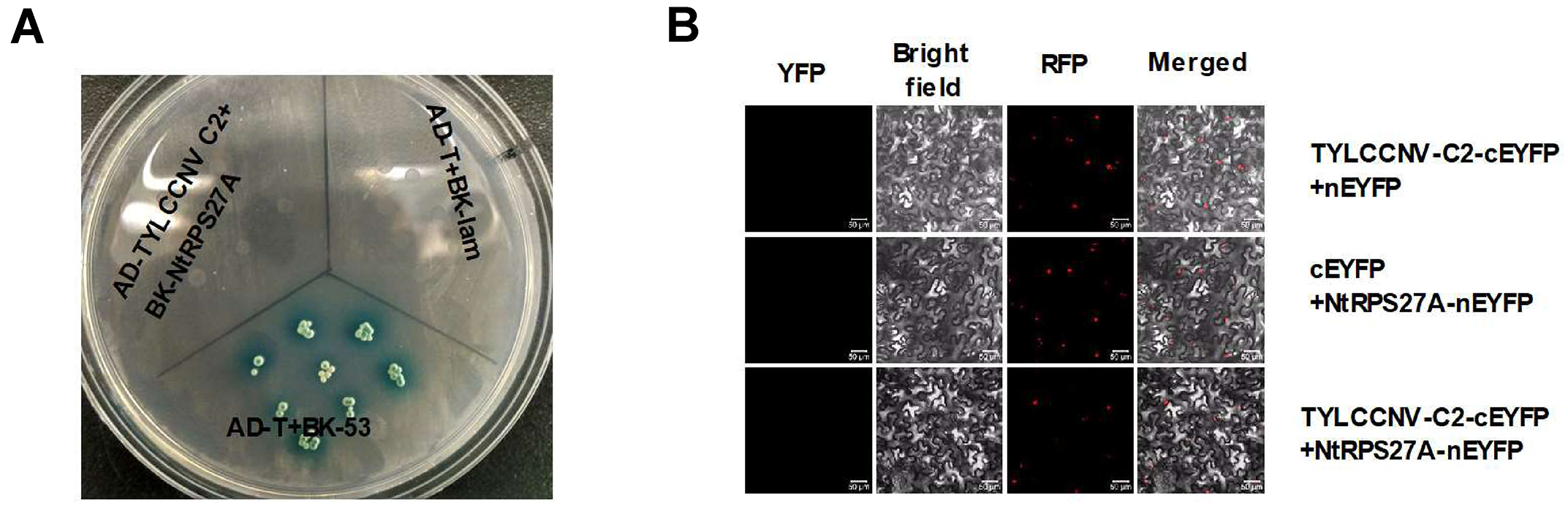

Supplement: S8 Fig — (A) Interaction between TYLCCNV-C2 and NtRPS27A in the yeast two-hybrid system. Yeast strain Y2H Gold co-transformed with the indicated plasmids was spotted on synthetic medium SD-Leu-Trp-His with x-α-gal and 2 mM 3-amino-1,2,4-triazole. The empty vectors pGBKT7 and pGADT7 were used as negative controls. (B) In vivo BiFC analysis of TYLCCNV-C2 interaction with NtRPS27A. No fluorescence signal was observed suggesting that TYLCCNV-C2 and NtRPS27A did not interact. Nuclei of tobacco leaf epidermal cells were marked with a RFP fusion protein. Bars = 50 mm. (TIF) [file ppat.1007607.s008.tif]

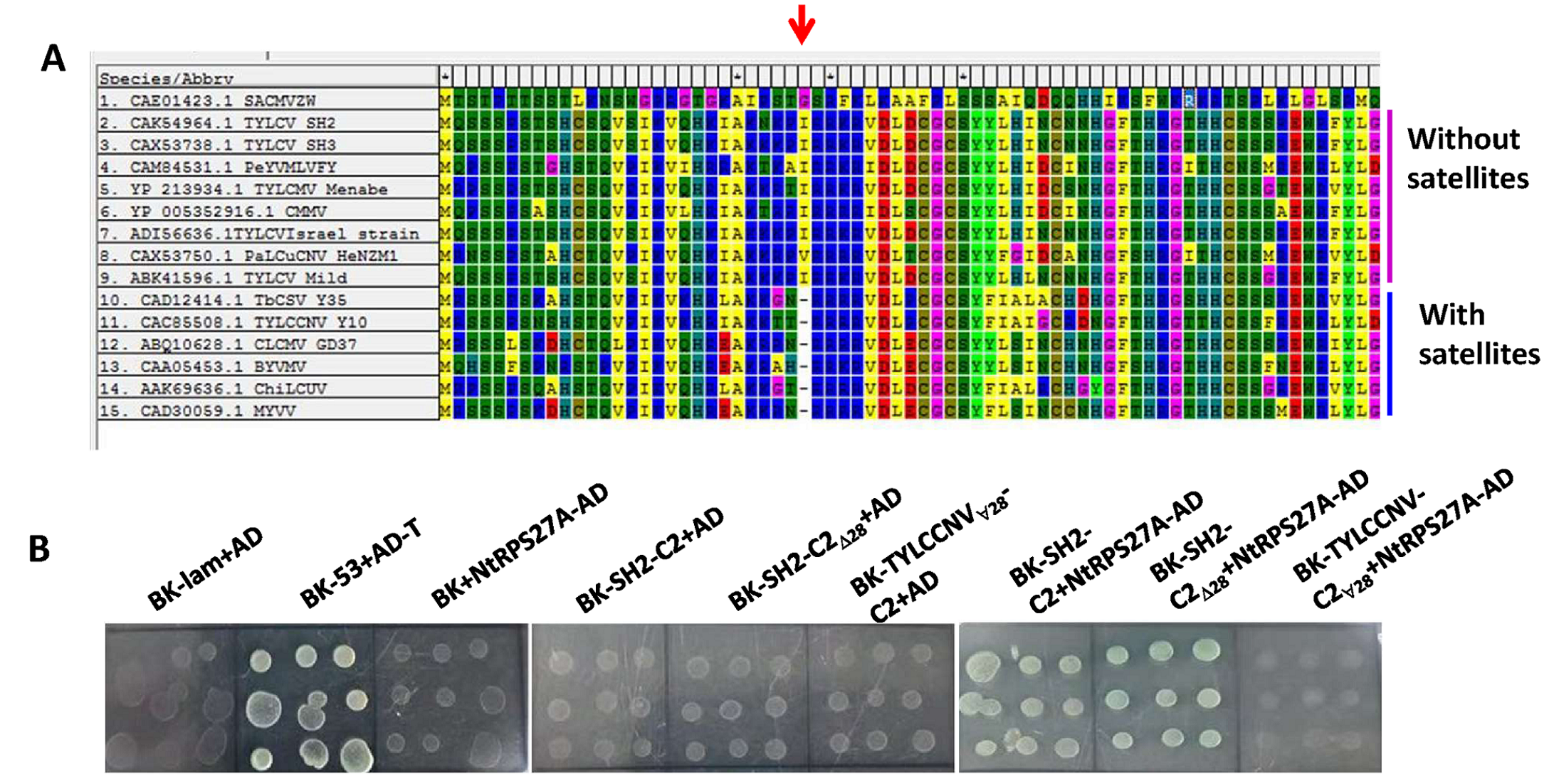

Supplement: S9 Fig — (A) Sequence alignment of C2 among different begomoviruses. The No. 1 virus is a bipartite virus; 2 to 9 are monopartite viruses not associated with satellites; 10 to 15 are monopartite viruses with satellites. (B) The 28th amino acid of C2 protein did not determine the interaction with NtRPS27A. TYLCCNV-C2∀28 indicates insertion of 28th amino acid into C2 protein of TYLCCNV; SH2-C2Δ28 indicates TYLCV SH2 C2 protein which has a 28th amino acid deletion. (TIF) [file ppat.1007607.s009.tif]

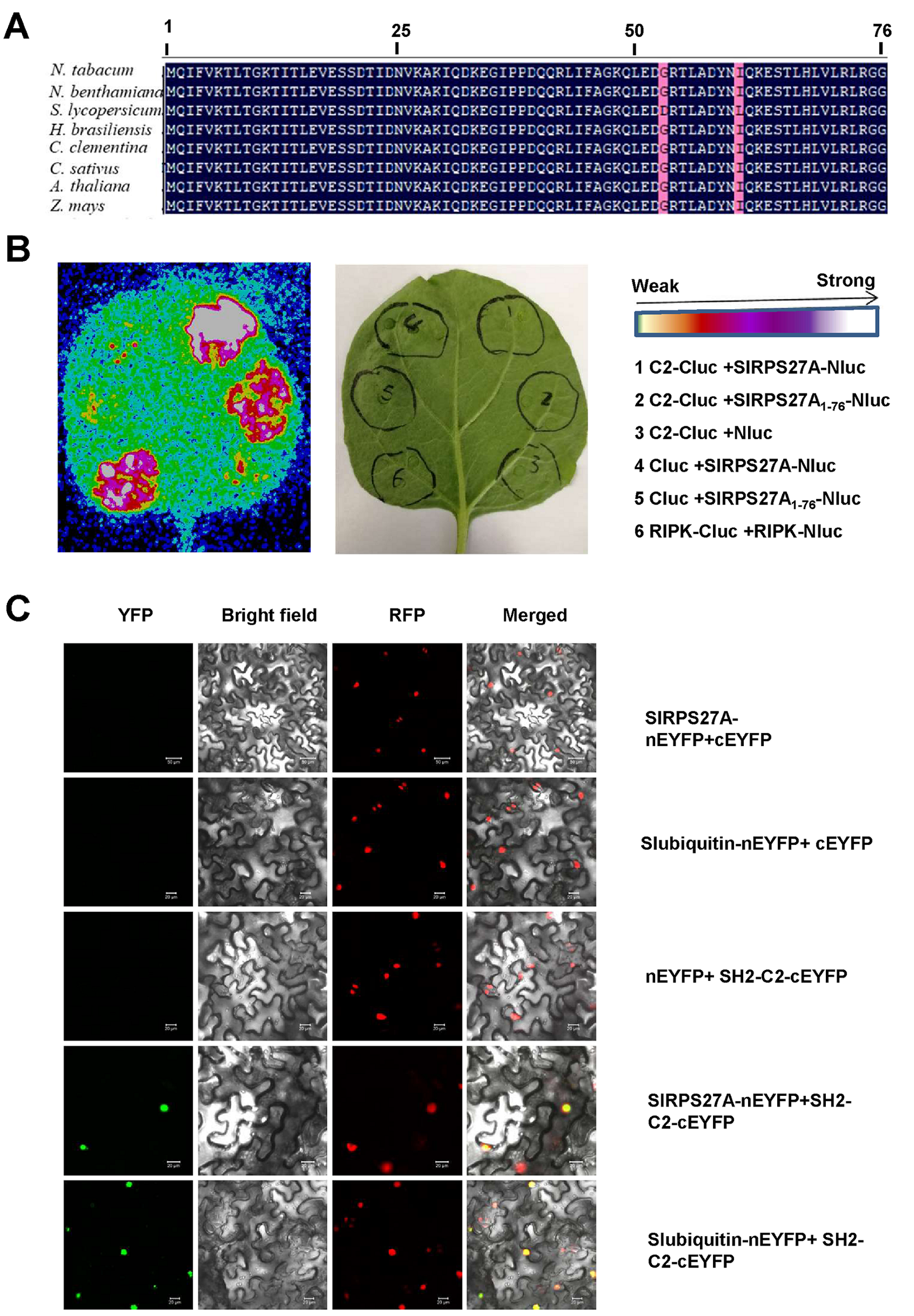

Supplement: S10 Fig — (A) Sequence alignments of ubiquitin moiety of RPS27A from different plants. (B) In vivo split-luciferase complementation assay of C2 interaction with SlRPS27A. (C) In vivo BiFC assay of C2 interaction with SlRPS27A. Nuclei of tobacco leaf epidermal cells were marked with a RFP fusion protein H2B-RFP. Bars = 20 mm or 50 mm. (TIF) [file ppat.1007607.s010.tif]
