# Supplementary material for: Plant begomoviruses subvert ubiquitination to suppress plant defenses against insect vectors
Source: PLoS Pathog. 2019 Feb 21;15(2):e1007607. doi: 10.1371/journal.ppat.1007607 (PMC6400417; doi:10.1371/journal.ppat.1007607)
Supplement: S1 Table — (DOCX) [file ppat.1007607.s011.docx]

**Table S1 Primers used in this article.**

| Usage | GenBank ID | Gene | sequence (5′→3′) |
| --- | --- | --- | --- |
| Transgene | AM282874 | TYLCV V2 F | TCCCCCGGGATGTGGGATCCACTTCT |
|  |  | TYLCV V2 R | TGCTCTAGATCAGGGCTTCGATACATT |
|  | AM282874 | TYLCV C2 F | TGCTCTAGAATGCAATCTTCGTCACCCT |
|  |  | TYLCV C2 R | AAAACTGCAGTCTAAATACTCTTAAGAAACG |
|  | AM282874 | TYLCV C4 F | CGGGGTACCATGGGGAACCACATCTCC |
|  |  | TYLCV C4 R | CGCGGATCCTTAATATATTGAGGGCCTCGG |
| BiFC | AM282874 | TYLCV C2 F | CCCTTAATTAACATGCAATCTTCGTCACCCT |
|  |  | TYLCV C2 R | GGGACTAGTAATACTCTTAAGAAACGAC |
|  | XM_016650758 | NtRPS27A F | CCCTTAATTAACATGCAGATCTTCGTGAAAAC |
|  |  | NtRPS27A R | GGGACTAGTGTCGGCGCCGGCCTTGTT |
|  |  | qutin 1-76 R | GGGACTAGTACCACCACGGAGACGGAGCAC |
|  |  | qutin 32-76 F | CCCTTAATTAACATGGATAAGGAAGGGATTCCCC |
|  | HM466976 | MYC2c R | ACTGCCACCTCCTCCACTAGTGCGTGTTTCAGCAACTCTGGATGTCAA |
|  |  | MYC2c F | ACGAACGATAGTTAATTAACATGACTGATTACAGCTTACCCACC |
|  | JQ172768 | JAZ1 F | ACGAACGATAGTTAATTAACATGGAGAGAGATTTTATGGG |
|  |  | JAZ1 R | ACTGCCACCTCCTCCACTAGTGGTCTCCTTACCGGCTATCAGA |
|  | NM_116090 | AtRPS27A F | CCCTTAATTAACATGCAGATCTTCGTGAAAAC |
|  |  | AtRPS27A R | GGGACTAGTAGCTTCAACTCCTTCTTTCTG |
|  | FN256260 | PAL C2 F | CCCTTAATTAACATGCAATCTTCGTCACCCTC |
|  |  | PAL C2 R | GGGACTAGTAATACTCTTAAGAAACGCCAAG |
| Prokaryotic Expression | XM_016650758 | RPS27A F | CGCGGATCCATGCAGATCTTCGTGAAAAC |
|  |  | RPS27A R | CCGCTCGAGTCAATCGGCGCCGGCCTTGT |
|  |  | quitin N R | CCGCTCGAGTCAACCACCACGGAGACGGAG |
|  |  | quitin 32-76 F | CCGGAATTCATGGATAAGGAAGGGATTCCCC |
|  | NM_116090 | AtRPS27A F | CGCGGATCCATGCAGATCTTCGTGAAAAC |
|  |  | AtRPS27A R | CCGCTCGAGTCAAGCTTCAACTCCTTCTTTCTG |
|  | AM282874 | TYLCV C2 F | GTCGACATGCAATCTTCGTCACCCT |
|  |  | TYLCV C2 R | GGATCCCTAAATACTCTTAAGAAACG |
|  | FN256260 | PAL C2 F | CGCGTCGACATGCGGAATTCATCACCCTC |
|  |  | PAL C2 R | CGCGGATCCCTAAATACTCTTAAGAAACG |
|  | JQ172768 | JAZ1 F | CGCGGATCCAATTTATTGCCAATGATTGA |
|  |  | JAZ1 R | CCGGAATTCTAAATCAGCAACAGAGGATT |
| qRT-PCR | XM_016643257 | GAPDH F | GCAGTGAACGACCCATTTATCTC |
|  |  | GAPDH R | AACCTTCTTGGCACCACCCT |
|  | HM466975 | MYC2b F | ATCGGATGGGATGCTATGA |
|  |  | MYC2b R | GAAGCTGCTCTTGCGTGTA |
|  | HM466976 | MYC2c F | AACCCTTCAACAGCGTCTT |
|  |  | MYC2c R | CCTTTGTAGTAACCATCTCCC |
|  | Y08847 | EAS F | TGCCGAGTTATTGATGATACAGC |
|  |  | EAS R | GCCATTCCTTGAAATTTAGCC |
|  | XM_016588402 | EAS12 F | CCTTGCGACAACATCGTATTT |
|  |  | EAS12 R | TCAACTTCGTATGTGGCTGTGT |
|  | AY528645 | cyclase F | TTTGCTGAAGGACTCGGT |
|  |  | cyclase R | TGCAGTAGTGGCTGGTGA |
|  | XM_016650758 | RPS27A F | GACCAGCAGCGGCTAATT |
|  |  | RPS27A R | CGGAGACGAAGCACCAAA |
|  | AB778304 | AOS F | TTTGCTAGATGGTAAAAGTTT |
|  |  | AOS R | CTGGTAGATCAGTGGATGG |
|  | AJ308487 | AOC F | GAACGTGACCGTGGTAGC |
|  |  | AOC R | CATAATCGCCGAAGTAGA |
|  | KF701476 | OPR3 F | GGTTGGAAGTGAAGAGGAG |
|  |  | OPR3 R | GGATTAGAAATGAAAAGGC |
|  | AB110952 | ADC1 F | ATCCTGTCCGCACATACCA |
|  |  | ADC1 R | GAACTTATCAATCTTCCCATCACTA |
|  | AF233849 | ODC F | GGCGTCTCATTCCACATC |
|  |  | ODC R | CGACATCCCGAGTTTAGC |
|  | AB038494 | QPT F | GCGATAAAGTTCATAAAGGC |
|  |  | QPT R | TCCAAGATGTAAGCAGGGT |
| Yeast two hybrid | AM282874 | TYLCV C2 F | CCGGAATTCATGCAATCTTCGTCACC |
|  |  | TYLCV C2 R | CGCGGATCCCTAAAATAGAGGGGATTG |
| Virus detection | AM282874 | TYLCV F | ATCGAAGCCCTGATATCCCCCGTGG |
|  |  | TYLCV R | CAGAGCAGTTGATCATG |
| Subcelluar Location | XM_016650758 | RPS27A F | TGCTCTAGAATGCAGATCTTCGTGAAAAC |
|  |  | RPS27A R | TCCCCCGGGATCGGCGCCGGCCTTGT |
|  |  | qutin N R | TCCCCCGGGACCACCACGGAGACGGAGCAC |
|  |  | qutin 32-76 F | TGCTCTAGAATGGATAAGGAAGGGATTCCCC |
|  | FN256260 | PAL C2 F | CGCTCTAGAATGCGGAATTCATCACCCTC |
|  |  | PAL C2 R | CGCGGATCCAATACTCTTAAGAAACG |
|  | NM_116090 | AtRPS27A F | CGCTCTAGAATGCAGATCTTCGTGAAAAC |
|  |  | AtRPS27A F | TCCCCCCGGGAGCTTCAACTCCTTCTTTCTG |
|  | AM282874 | TYLCV C2 F | TGCTCTAGAATGCAATCTTCGTCACCCT |
|  |  | TYLCV C2 R | TCCCCCGGGAATACTCTTAAGAAACGAC |
| VIGS | HM466975 | MYC2 F | CGCGGATCCCTTGGTGATGCAATTGCATT |
|  |  | MYC2 R | TGCTCTAGACCCATTTTCACTGTCGCTTG |
|  | AY528645 | cyclase F | TGCTCTAGAACAGTACTGCTTCATCAATG |
|  |  | cyclase R | CGCGGATCCTAGATCCAAATTAAGTTT |
|  | XM_016650758 | RPS27A F | TGCTCTAGACATTTGGTGCTTCGTCTC |
|  |  | RPS27A R | CGCGGATCCAAGTGGTTAGCCATGAAAG |
|  | AF233849.1 | ODC F | TGCTCTAGACAATCATCGTTTCCGGGTT |
|  |  | ODC R | CGCGGATCCGCATGGATTTGCGAAAACAA |
